# Dissociations in the Effects of β_2_-Adrenergic Receptor Agonists on cAMP Formation and Superoxide Production in Human Neutrophils: Support for the Concept of Functional Selectivity

**DOI:** 10.1371/journal.pone.0064556

**Published:** 2013-05-31

**Authors:** Irena Brunskole Hummel, Michael T. Reinartz, Solveig Kälble, Heike Burhenne, Frank Schwede, Armin Buschauer, Roland Seifert

**Affiliations:** 1 Institute of Pharmacology, Medical School of Hannover, Hannover, Germany; 2 Department of Pharmaceutical and Medicinal Chemistry II, University of Regensburg, Regensburg, Germany; 3 Biolog Life Science Institute, Bremen, Germany; China Medical University, Taiwan

## Abstract

In neutrophils, activation of the β_2_-adrenergic receptor (β_2_AR), a G_s_-coupled receptor, inhibits inflammatory responses, which could be therapeutically exploited. The aim of this study was to evaluate the effects of various β_2_AR ligands on adenosine-3′,5′-cyclic monophosphate (cAMP) accumulation and N-formyl-L-methionyl-L-leucyl-L-phenylalanine (fMLP)-induced superoxide anion (O_2_^•−^) production in human neutrophils and to probe the concept of ligand-specific receptor conformations (also referred to as functional selectivity or biased signaling) in a native cell system. This is an important question because so far, evidence for functional selectivity has been predominantly obtained with recombinant systems, due to the inherent difficulties to genetically manipulate human native cells. cAMP concentration was determined by HPLC/tandem mass spectrometry, and O_2_^•−^ formation was assessed by superoxide dismutase-inhibitable reduction of ferricytochrome c. β_2_AR agonists were generally more potent in inhibiting fMLP-induced O_2_^•−^ production than in stimulating cAMP accumulation. (−)-Ephedrine and dichloroisoproterenol were devoid of any agonistic activity in the cAMP assay, but partially inhibited fMLP-induced O_2_^•−^ production. Moreover, (−)-adrenaline was equi-efficacious in both assays whereas the efficacy of salbutamol was more than two-fold higher in the O_2_^•−^ assay. Functional selectivity was visualized by deviations of ligand potencies and efficacies from linear correlations for various parameters. We obtained no evidence for involvement of protein kinase A in the inhibition of fMLP-induced O_2_^•−^ production after β_2_AR-stimulation although cAMP-increasing substances inhibited O_2_^•−^ production. Taken together, our data corroborate the concept of ligand-specific receptor conformations with unique signaling capabilities in native human cells and suggest that the β_2_AR inhibits O_2_^•−^ production in a cAMP-independent manner.

## Introduction

Human neutrophils are crucial for the defense of the host organism against infectious agents such as bacteria, fungi, protozoa, viruses and tumor cells. After phagocytosis of invading agents neutrophils are able to destruct them, the respiratory burst NADPH oxidase being a major player [1]. This enzyme catalyzes the univalent reduction of molecular oxygen (O_2_) to the superoxide anion (O_2_^•−^) with NADPH as electron donor [2]–[5]. Activation of neutrophils is triggered by bacterial formyl peptides [6]. Upon binding of N-formyl-L-methionyl-L-leucyl-L-phenylalanine (fMLP) to the formyl peptide receptor, which is G_i_-coupled [7]–[8], O_2_^•−^ production in neutrophils increases [1]. fMLP-stimulated O_2_^•−^ production in neutrophils is counteracted by compounds that increase the intracellular adenosine-3′,5′-cyclic monophosphate (cAMP) concentration [2]. These compounds include prostaglandins, the inhibitor of phosphodiesterases, 3-isobutyl-1-methylxanthine (IBMX), membrane-permeable analogs of cAMP as well as agonists of the β_2_-adrenergic receptor (β_2_AR) [9]–[14]. Furthermore, fMLP-stimulated O_2_^•−^ formation is enhanced by the incubation of neutrophils with *N*-(2-{[(*E*)-3-(4-bromophenyl)prop-2-enyl]amino}ethyl)isoquinoline-5-sulfonamide (H-89), an inhibitor of cAMP-dependent protein kinase (PKA) [11]. Canonically, the β_2_AR couples to G_s_ proteins in order to activate adenylyl cyclases (AC) resulting in increased intracellular cAMP concentration [2]. Nevertheless, the β_2_AR can also couple to G_i_ proteins, G_q_ proteins and β-arrestin, triggering responses distinct from those activated through G_s_ proteins [15]–[19].

Classic models of G-protein-coupled receptor (GPCR) activation postulate the existence of a single active (R*) and an inactive (R) state [20]–[22]. In the active R* state, the receptor is assumed to activate its cognate G protein and regulate down-stream effectors. However, over the past 15–20 years, compelling evidence from various groups has accumulated that the R/R* dichotomy is too simplicistic. These studies comprise biochemical, pharmacological and biophysical approaches [19]–[39]. Accordingly, it is now generally assumed that any given ligand stabilizes a ligand-specific receptor conformation with unique signaling capabilities, resulting in ligand-specific activation of G-proteins and/or β-arrestin [27], [28]. Stabilization of ligand-specific receptor conformations with unique signaling capabilities is also referred to as functional selectivity or biased signaling [20]–[22], [29]–[33]. To this end, most of the evidence for ligand-specific receptor conformations has been obtained in studies with recombinant systems [34]–[36] or purified receptor proteins [31], [37]–[39], but studies on the relevance of ligand-specific receptor conformations in native human cells are still largely missing. Reasons for this lack of knowledge are inherent difficulties to manipulate human cells genetically. In addition, after isolation, human blood cells such as neutrophils survive only for limited period of time [40]. However, since the concept of functional selectivity implies that certain ligands can be clinically more efficacious in a given setting while displaying less unwanted effects, it is of paramount importance to probe functional selectivity in native cells.

Recently, we have reported on the functional selectivity of another G_s_-coupled receptor, the histamine H_2_ receptor, in two native cell systems, human eosinophils and neutrophils [40]. The pharmacological profiles of H_2_R agonists as well as H_2_R antagonists do not match by comparing their effects on eosinophils and neutrophils as well as by comparing these parameters with data obtained in a recombinant test system. Moreover, even in the same cell type, differences were observed when ligands were characterized determining two different parameters. Each ligand triggers unique effects depending on the test system and parameters measured which is of importance for further drug development.

The aim of the present study was the characterization of the β_2_AR on human neutrophils with a series of structurally diverse β_2_AR ligands and thereby, to probe the concept of ligand-specific receptor conformations on one of the most important and best-characterized GPCRs in a physiologically relevant native human cell system. Two distinct parameters were chosen for the characterization of the β_2_AR on neutrophils. The first parameter was measurement of the cAMP content in neutrophils. The second parameter was monitoring of the β_2_AR-mediated effects on fMLP-stimulated O_2_^•−^ production. We also examined the effects of various pharmacological tools including protein kinase inhibitors, AC inhibitors and activators and various cAMP analogues on fMLP-induced O_2_^•−^ production in order to obtain further insights into the mechanisms underlying inhibition of NADPH oxidase.

## Materials and Methods

### Materials

(S*,S*)-3-(Isopropylamino)-1-(7-methyl-2,3-dihydro-1*H*-inden-4-yloxy)butan-2-ol) ((±)-(S*, S*)-ICI 118551) (ICI) and (±)-bisoprolol (BIS) were obtained from Tocris Bioscience (Avonmouth, Bristol, UK). (−)-Isoproterenol (ISO), (−)-adrenaline (ADR), (±)-salbutamol (SAL), (±)-dobutamine (DOB), (±)-metoprolol (MET), (±)-alprenolol (ALP), (±)-atenolol (ATE) and forskolin (FSK) were from Sigma-Aldrich (St. Louis, MO, USA). (−)-Ephedrine (EPH) was from Mallinckrodt (St. Louis, MO, USA) and (±)-dichlorisoproterenol (DCI) from Aldrich (Milwaukee, WI, USA). Chemical structures of ligands are depicted in Fig. S1. Stock solutions of ISO, ADR, SAL, DOB, EPH and DCI (10 mM each) were prepared in 1 mM HCl and stock solutions of ICI, MET, ALP, BIS and ATE in Millipore water. Dilution series of all ligands were prepared in Millipore water. Dulbecco’s PBS (DPBS, 10 x) without Ca^2+^ and Mg^2+^ (pH 6.5–7.0) was purchased from PAN Biotech (Aidenbach, Germany) and Biocoll separating solution from Biochrom (Berlin, Germany). Trypan blue solution, ferricytochrome c, cytochalasin B, fMLP and IBMX were from Sigma-Aldrich. Solvents for extraction and HPLC analysis were purchased as follows: HPLC-gradient grade water and methanol from J. T. Baker (Deventer, The Netherlands), ammonium acetate from Sigma-Aldrich and acetic acid from Riedel-de Haen (Hannover-Seelze, Germany). Tenofovir was obtained from the National Institutes of Health (Bethesda, MD, USA). N^6^,2′-O-dibutyryladenosine-3′,5′-cyclic monophosphate (DB-cAMP) (>96%; <3% monobutyryl derivatives; <0.5% cAMP) was purchased from Sigma-Aldrich. cAMP (>99.9%), adenosine-3′,5′-cyclic monophosphorothioate, Rp-isomer (Rp-cAMPS) (>99.94%), N^6^-monobutyryladenosine-3′,5′-cyclic monophosphate (6-MB-cAMP) (>99.56%), 2′-O-monobutyryladenosine-3′,5′-cyclic monophosphate (2′-O-MB-cAMP) (>99.69%), adenosine-3′,5′-cyclic monophosphorothioate, Sp-isomer (Sp-cAMPS) (>99.96%), and 8-(4-chlorophenylthio)-2′-O-methyladenosine-3′,5′-cyclic monophosphate (8-pCPT-2′-O-Me-cAMP) (>99.97%) were obtained from BIOLOG Life Science Institute (Bremen, Germany). Structures of cAMP analogs are shown in Fig. S2. Purities of cyclic nucleotides were determined by HPLC. Stock solutions of nucleotides (100 mM each) were prepared with Millipore water. H89 was obtained from Merck (Darmstadt, Germany) as a 10 mM solution in DMSO. KT5720 was from Enzo Life Sciences (Farmingdale, NY, USA) and a 10 mM stock solution was prepared in DMSO. SQ 22536 (Merck, Darmstadt, Germany) was prepared as a 10 mM solution in DMSO. FSK (10 mM) was dissolved in DMSO as well. Phorbol-12-myristate-13-acetate was prepared as a 10 mM solution in DMSO and was purchased from Sigma. Working solutions of all named substances were prepared by diluting stock solution with Millipore water.

### Isolation of Human Neutrophils

This study and the consent procedure were approved by the Ethics Committee of the Medical School of Hannover. Written consent was obtained by all volunteers. The completed and signed consent forms are kept on file in the secretary of the Institute of Pharmacology of the Medical School of Hannover. Human neutrophils were isolated from venous blood of healthy volunteers of either sex (1.6 mg EDTA/ml blood as anticoagulant) or from buffy coat obtained from the Institute for Transfusion Medicine (Medical School of Hannover, Germany). Buffy coat preparations were also obtained from individual donors. All isolation steps were carried out at room temperature. Firstly, 7 ml of venous blood or 5 ml of buffy coat were diluted to 35 ml with 1×DPBS and carefully layered onto 15 ml of Biocoll separating solution (density 1.077 g/ml) in a 50 ml-Falcon tube. Following centrifugation (30 min, 400×g), the upper three layers were removed. The residual pellet (∼2 ml), which contained erythrocytes and granulocytes, was resuspended in 18 ml of Millipore water and incubated for 1 min under gentle agitation in order to achieve selective lysis of erythrocytes. Afterwards, isotonicity was restored by adding 2.2 ml of 10×DPBS, and centrifugation at 300×g for 5 min followed. The lysis step was repeated once to remove residual erythrocytes. The cell pellet was re-suspended in 5 ml of 1×DPBS and sedimented by centrifugation at 300×g for 5 min. The resulting cell preparation consisted of viable neutrophils (>98%), as assessed by the trypan blue exclusion test. Finally, neutrophils were suspended in 1×PBS (1×10^6^ cells/ml for the O_2_^•−^ assay or 1×10^7^ cells/ml for the determination of cAMP) and stored on ice until use. Experiments were performed within 4 h after completion of isolation because at later time points, viability of cells declined substantially as assessed by trypan blue dye uptake and declined responsiveness to receptor ligands (data not shown).

### Superoxide Anion Generation (O_2_^•−^ Assay)

Reactions were carried out in 96-well plates in triplicate. Standard reaction mixtures (total volume 200 µl) contained 1 mM CaCl_2_, 100 µM ferricytochrome c, 0.3 µg/ml cytochalasin B (priming role by enhancing O_2_^•−^ formation upon exposure to fMLP) [2], ligands at different concentrations (where indicated, additionally PKA inhibitors, AC inhibitors or cAMP analogs) and 1×10^5^ neutrophils in 1×DPBS. After pre-incubation of the reaction mixtures for 3 min at 37°C, reactions were initiated by addition of fMLP (1 µM). Reference samples contained all components listed above except for fMLP. O_2_^•−^ formation was continuously measured by monitoring the reduction of ferricytochrome c at 550 nm for 30 min at 37°C, using a Synergy 4 microplate reader (BioTek Instruments, Winooski, VT, USA). The difference in absorbance at 550 nm between 0 min (addition of fMLP) and 30 min was used for subsequent data analysis, in order to assess agonistic activity of examined ligands. With the exception of DOB, all examined test compounds did neither reduce ferricytochrome c nor stimulate O_2_^•−^ production *per se* nor acted as radical scavenger as assessed by the lack of effect on phorbol ester-stimulated O_2_^•−^ production (data not shown). As at DOB concentrations higher than 500 nM, ferricytochrome c reduction took place, the maximum concentration of DOB used in the O_2_^•−^ assays was 500 nM.

### cAMP Accumulation and Extraction from Neutrophils (cAMP Assay)

Reactions were conducted in triplicate in 1.5 ml Eppendorf reaction vessels in a total volume of 100 µl. Fifty µl of the reaction mixture containing CaCl_2_ (1 mM final concentration after addition of neutrophils), IBMX (non-selective phosphodiesterase inhibitor; 100 µM) and the respective ligand at different concentrations in 1 x DPBS were pre-incubated for 5 min at 37°C. Isolated neutrophils suspended in 1×DPBS were pre-incubated separately for 10 min at 37°C. Following the addition of 50 µl of neutrophils (5×10^5^ cells/reaction vessel) to reaction mixture, samples were incubated for 10 min at 37°C. Afterwards, samples were incubated for 10 min at 95°C in order to denature cell proteins and then cooled to 4°C. One hundred µl of ice-cold internal standard (tenofovir; 100 ng/ml) in eluent A (3/97 MeOH/H_2_O, 50 mM NH_4_OAc, 0.1% HOAc) were added. The suspension was centrifuged at 20.800×g at 4°C for 5 min in order to remove denatured proteins. The cAMP concentration of the supernatant was determined by reversed phase HPLC coupled to mass spectrometry (HPLC-MS/MS).

### Quantitation of cAMP by HPLC-MS/MS

In this study, cAMP levels were determined by HPLC-MS/MS which is characterized by extremely high sensitivity and specificity [41]–[42]. Since this method is not yet commonly known and used, we describe the experimental protocol in some detail. The chromatographic separation was performed on an Agilent 1100 Series HPLC System (Agilent Technologies, Santa Clara, CA, USA) equipped with a binary pump system and with a 100 µl sample loop. A combination of Supelco Column Saver (2.0 µm filter, Supelco Analytical, Bellafonte, CA, USA), Security Guard Cartridge (C18, 4×2 mm) in an Analytical Guard Holder KJO-4282 (Phenomenex, Aschaffenburg, Germany) and an analytical Zorbax Eclipse XDB-C16 column (50×4.6 mm, 1.8 µm particle size, Agilent Technologies), temperature controlled by a HPLC column oven at 25°C, were used. The binary pump system supplied eluent A (50 mM ammonium acetate and 0.1% (v/v) acetic acid in a methanol/water mixture (3/97 (v/v)) and eluent B (50 mM ammonium acetate and 0.1% (v/v) acetic acid in a methanol/water mixture (97/3 (v/v)). The injection volume was 50 µl and the flow rate of 0.4 ml/min remained constant throughout the chromatographic run. From 0 to 5 min, the gradient of eluent B was linearly increased from 0 to 50% of eluent B, and re-equilibrium of the column to 100% of eluent A was achieved from 5 to 8 min. Retention times of the analyte cAMP and the internal standard tenofovir were 6.2 and 5.4 min, respectively. The internal standard was used to mathematically correct the loss of cAMP during preparation as well as possible variabilities in HPLC-MS/MS measurement. Analyte detection was conducted on an AB Sciex QTRAP 5500 triple quadrupole mass spectrometer (AB Sciex, Foster City, CA, USA) using selected reaction monitoring (SRM) analysis in positive ionization mode. For this purpose nitrogen was used as collision gas. Using a 50 ms dwell time, SRM transitions were monitored as follows: cAMP +330/136 and +330/312, tenofovir +288/176 and +288/159. The transition +330/136 was the most intense transition of cAMP and therefore used for quantification. Additionally the +330/312 transition of cAMP was used as qualifier. The transition +288/176 of tenofovir was used as quantifier and the transition +288/159 as qualifier. The mass spectrometer parameters were as follows: ion source voltage: 4500 V, ion source temperature: 600°C, curtain gas: 30 psi and collision gas: 9 psi. cAMP in samples was quantified by applying the standard curve, obtained by analysis of known amounts of pure cAMP at: 0.0262, 0.066, 0.164, 0.41, 1.024, 2.56, 6.4, 16, 40, 100, 250 pmol/tube.

### Miscellaneous (Data Analysis, Statistical Analysis and GTPase Assay)

Chromatograms, obtained by the HPLC-MS/MS analysis, were analyzed with the Analyst Software 1.5.1 (AB Sciex). Steady-state GTPase activity assay, using membrane preparations of Sf9 insect cells, expressing fusion protein β_2_AR-G_sαS_, was performed as described previously [43]. Data from the O_2_^•−^, cAMP and GTPase assays were analyzed with the Prism 5.01 software (GraphPad, San Diego, CA, USA). The means ± S.E.M. were always determined by the analysis of at least three independent experiments, performed in triplicate, if not indicated otherwise.

The efficacy (*E*_max_) of ISO in each assay was set to 1.00 and the efficacies of other ligands were referred to this value. The p*K*_B_ values for β_2_AR antagonists were calculated according to Cheng and Prusoff [44] using the following equation: p*K*_B_ = - log(IC_50*antagonist*_/(1+(c*_ISO_*/x))); IC_50*antagonist*_ – IC_50_ value of an antagonist, determined in antagonist mode, c*_ISO_* – used concentration of ISO, ×– IC_50_ (O_2_^•−^ assay) or EC_50_ value (cAMP assay and GTPase assay) for ISO, determined in agonist mode (data from Table 1).

**Table 1:** Comparison of potencies and efficacies of standard β_2_AR agonists, determined in three different test systems.

Cpd.	O_2_^•−^ assay (β_2_AR on neutrophil granulocytes)		cAMP assay (β_2_AR on neutrophil granulocytes)		GTPase assay (recombinant protein hβ_2_AR-G_sαS_)	
	pIC_50_ *±* S.E.M. *(IC_50_ in µM)*	*E*_max_ *±* S.E.M.	pEC_50_ *±* S.E.M. *(EC_50_ in µM)*	*E*_max_ *±* S.E.M.	pEC_50_ *±* S.E.M. *(EC_50_ in µM)*	*E*_max_ *±* S.E.M.
**ISO**	8.02±0.07 *(0.0096)*	1.00	7.42±0.10 *(0.038)*	1.00	7.50**^b^** *(0.032)*	1.00^b,c^
**ADR**	7.82±0.09 *(0.015)*	1.01±0.05	6.81±0.09 *(0.16)*	1.06±0.04	7.37^c^ *(0.043)*	1.00^c^
**SAL**	7.16±0.12 *(0.069)*	0.77±0.04	6.74±0.15 *(0.18)*	0.35±0.03	6.70**^b^** *(0.20)*	0.74**^b^**
**DOB**	n.d.	0.15±0.03^a^	4.86±0.36 *(13.8)*	0.21±0.05	6.70**^b^** *(0.20)*	0.45**^b^**
**EPH**	5.95±0.23 *(1.12)*	0.34±0.04	<4 *(*<*100)*	0.01±0.01	4.69**^b^** *(20.5)*	0.31**^b^**
**DCI**	4.40±0.26 *(39.8)*	0.32±0.06	<4 *(*<*100)*	0.00±0.01	7.09**^b^** *(0.082)*	0.17**^b^**

On human neutrophil granulocytes, the O_2_^•−^ assay (1×10^5^ cells per well) and the cAMP assay (5×10^5^ cells per cup) were performed as described under *Materials and Methods*. Data were analyzed by non-linear regression and were best fitted to sigmoidal concentration/response curves. Data shown are the means of four to nine independent experiments performed in triplicate. The efficacy (*E*_max_) of ISO in each assay was set to 1.00 and the efficacies of other ligands were referred to this value. n.d. not determined. As DOB at concentrations higher than 500 nM caused reduction of ferricytochrome c *per se*, the reliable determination of pIC_50_ value was not possible. ^a^ Efficacy at concentration of 500 nM was taken as *E*_max_. ^b^ The data were taken from [45]. The reported non-logarithmic EC_50_ values were converted into logarithmic pEC_50_ values. ^c^ The data were taken from [43]. The non-logarithmic EC_50_ values shown in µM in parentheses were converted into logarithmic pEC_50_ value. The literature data on GTPase assay are lacking S.E.M., because the original data are represented in non-logarithmic manner and/or S.D. or 95% confidence interval is indicated instead of S.E.M. Therefore, calculation of S.E.M. from available data was impossible.

Data for receptor ligands were analyzed using one-way ANOVA, followed by Bonferroni’s multiple comparison test, in order to compare p*K*_B_ values of the examined β_2_AR antagonists between the O_2_^•−^, cAMP and GTPase assay. Data for AC- and PKA inhibitors were analyzed using one-way ANOVA, followed by Dunnett’s multiple comparison test. Statistical significance was defined as p < 0.05 (95% confidence interval).

## Results

### Characterization of the β_2_AR on Human Neutrophils with β_2_AR Agonists

**Figure 1:**
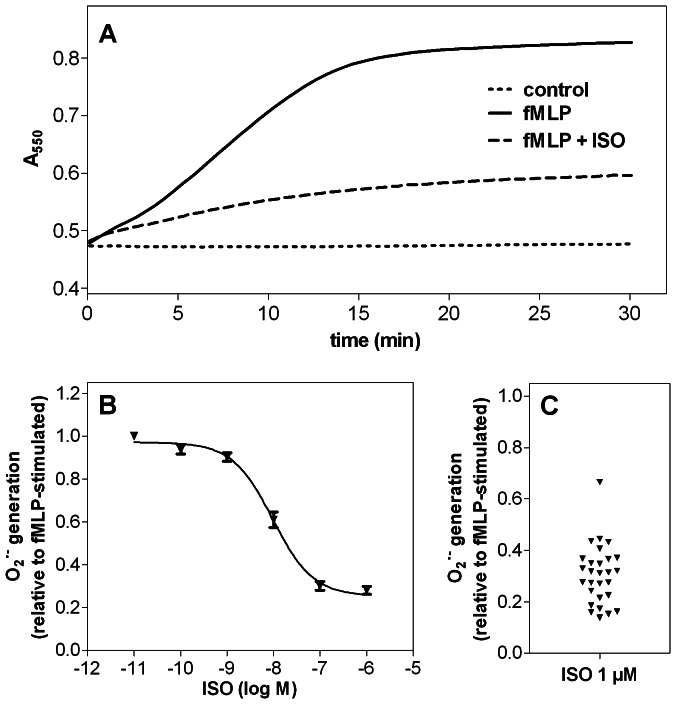
Superoxide anion generation assay (O_2_^•^^−^ assay). The O_2_^•−^ production in human neutrophil granulocytes (1×10^5^ cells per well) was monitored by measuring the superoxide dismutase-inhibitable reduction of ferricytochrome c at 550 nm as described under *Materials and Methods*. (**A**) Continuous measurement of O_2_^•−^ production for 30 min under control conditions (control), after stimulation with 1 µM fMLP (fMLP) and in the presence of 1 µM fMLP in combination with 1 µM ISO (fMLP+ISO). Data shown are from one representative experiment performed in triplicate. (**B**) Concentration-response curve for ISO in the O_2_^•−^ assay. Data shown are from nine independent experiments, performed in triplicate (data points are means ± S.E.M.). Data were analyzed by non-linear regression and were best fitted to sigmoidal concentration/response curve. (**C**) Inter-experimental variability of inhibitory effect of 1 µM ISO on fMLP-stimulated O_2_^•−^ production. Each data point represents one independent experiment. Increase in absorbance at 550 nm during 30 min after addition of fMLP was set to 1.00 and increase in absorbance in the presence of 1 µM ISO (+ fMLP) in each assay was compared to this value.

**Figure 2:**
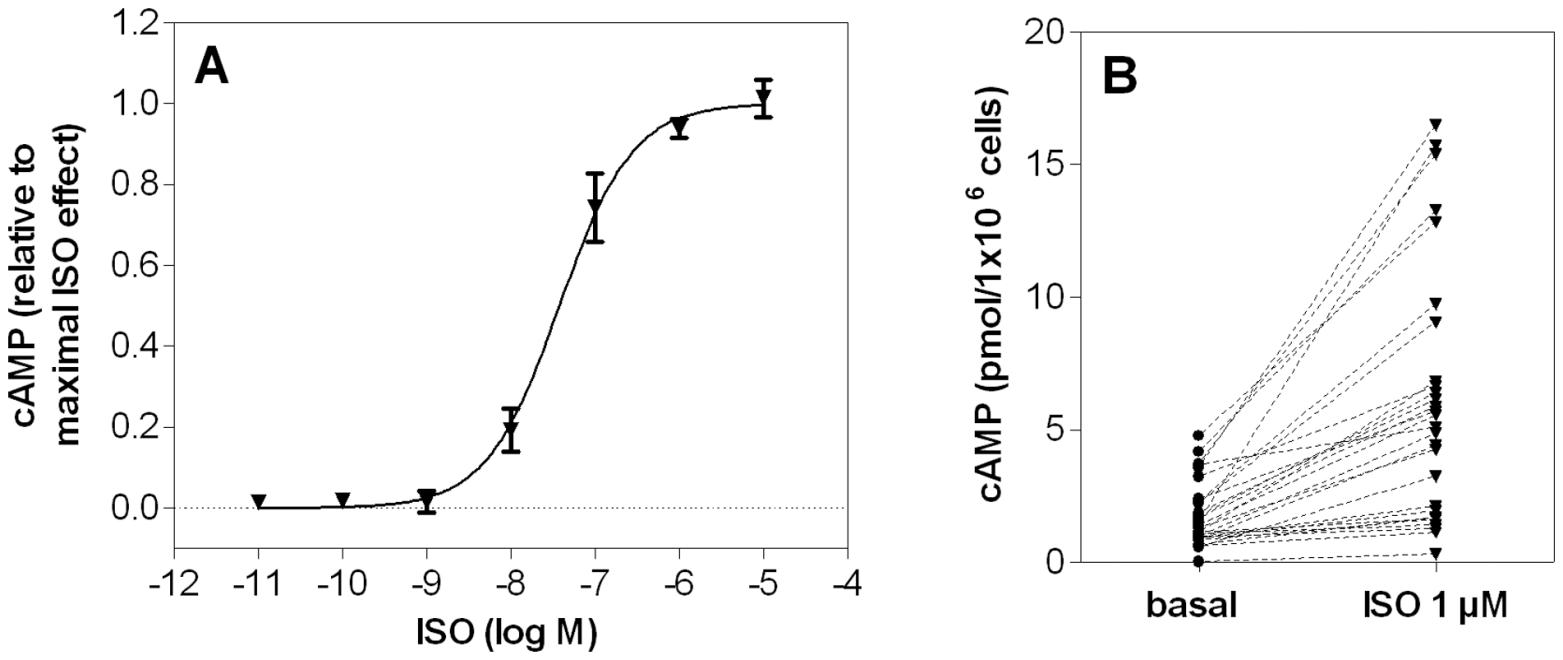
Measurement of cAMP content in neutrophil granulocytes (cAMP assay). cAMP accumulation in human neutrophil granulocytes (5×10^5^ cells per sample) was monitored by HPLC-MS/MS system as described under *Materials and Methods*. (**A**) Concentration-response curve for ISO in the cAMP assay. Data shown are from four independent experiments, performed in triplicate (data points are mean ± S.E.M.). The maximal ISO-induced cAMP production was set to 1.00. Data were analyzed by non-linear regression and were best fitted to sigmoidal concentration/response curve. **(B**) Inter-experimental variability of basal cAMP concentration in neutrophil granulocytes (basal) and cAMP level after stimulation with 1 µM ISO (ISO 1 µM). Each data point represents one independent experiment. cAMP levels after stimulation with ISO increased by 30–1000% relative to basal cAMP levels.

β_2_AR agonists with efficacies varying from very weak partial to full agonism [45] were examined. The effects of β_2_AR agonists were measured as inhibition of fMLP-stimulated O_2_^•−^ production (Fig. 1) and as cAMP accumulation (Fig. 2). In both cases, the inter-experimental variability was high (Figs. 1 and 2). Note that in Figs. 1C and 2B, data from different individuals are depicted. High inter-individual variability of human neutrophil function was observed previously [46]. However, when neutrophils from a given donor were analyzed on different days, data in the two test systems generally varied by less than 20% (data not shown). Thus, interindividual data variability is a much greater issue with neutrophils than intraindividual day-to-day variability. Accordingly, in order to allow comparison of results from experiments with different donors in this study, data were normalized with 1.00 representing the maximal effect of the β_2_AR agonist ISO and 0.00 representing the basal activity.

**Figure 3:**
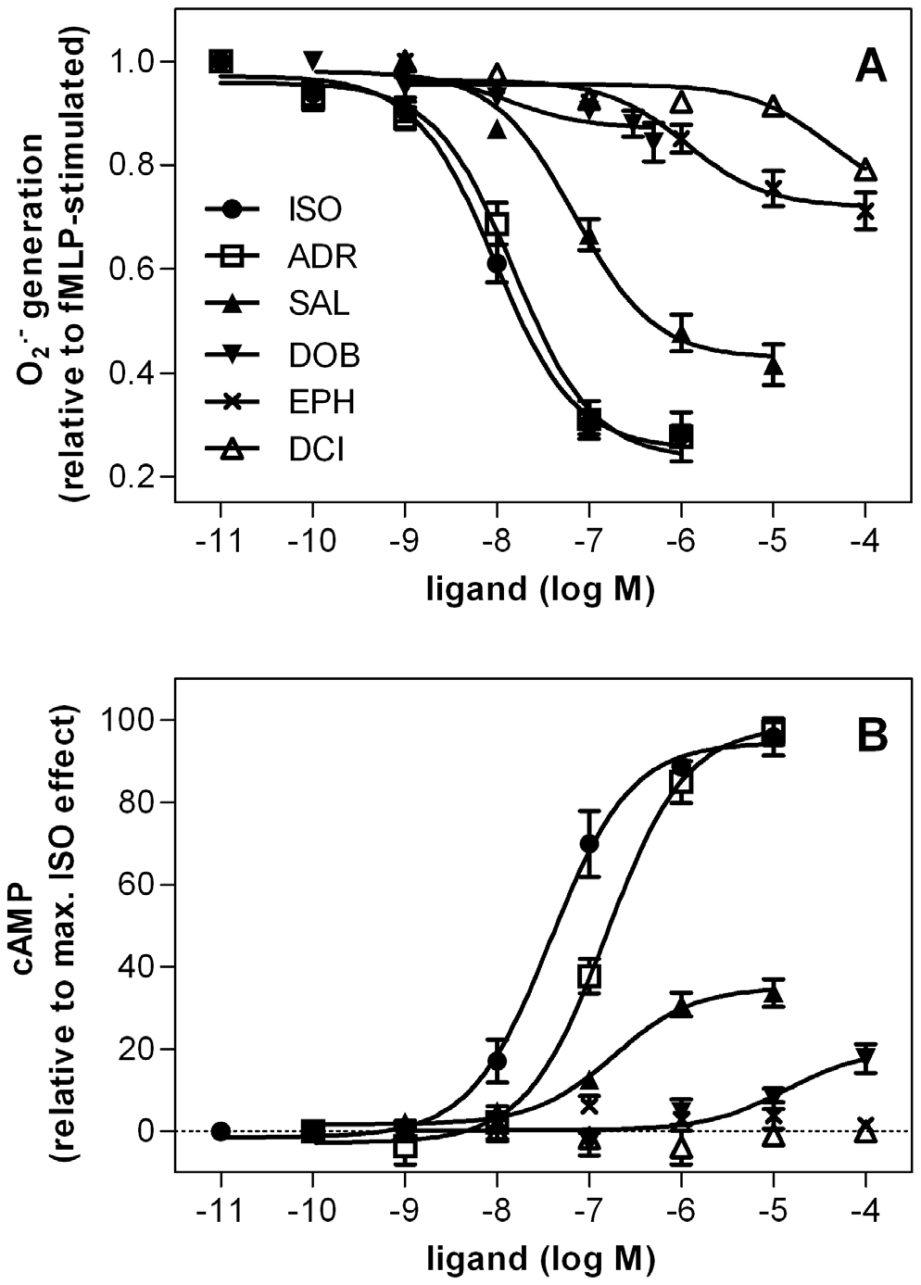
Concentration-response curves for β_2_AR agonists determined in the O_2_^•^^−^ assay (A) and cAMP assay (B). The O_2_^•−^ assay (1×10^5^ cells per well) and the cAMP assay (5×10^5^ cells per cup) were performed as described in *Materials and Methods*. Data were analyzed by non-linear regression and were best fitted to sigmoidal concentration/response curves. Data shown are the means ± S.E.M. of four to nine independent experiments performed in triplicate. As DOB at concentrations higher than 500 nM caused reduction of ferricytochrome c *per se*, the maximal DOB concentration examined in the O_2_^•−^ assay was 500 nM.

**Figure 4:**
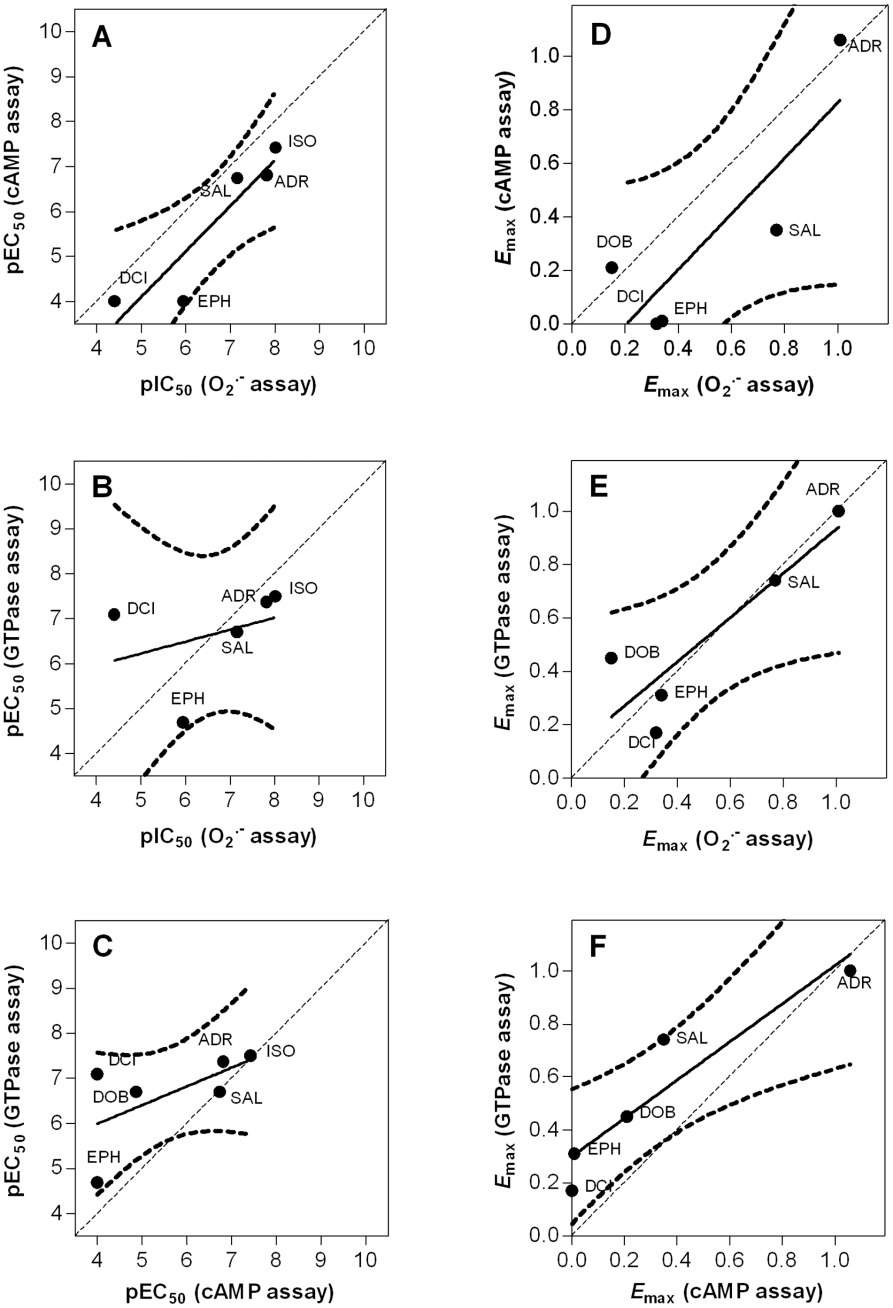
Pair-wise comparison of the potencies (A–C) and efficacies (D–F) of the β_2_AR agonists in the O_2_^•^^−^ assay, the cAMP assay and the GTPase assay. The data for comparison were taken from Table 1 and were analyzed by linear regression. The dashed lines represent the 95% confidence intervals in the regression line. The diagonal dotted line indicates a theoretical line for identical values (slope = 1). Slopes (95% confidence interval) and r^2^ of the calculated correlations are as follows; **A.** 1.02 (0.22 to 1.81), 0.85; **B.** 0.27 (−1.05 to 1.58), 0.12; **C.** 0.42 (−0.32 to 1.15), 0.38; **D.** 1.04 (−0.14 to 2.21), 0.73; **E.** 0.83 (0.02 to 1.63), 0.78; **F.** 0.72 (0.22 to 1.22), 0.89.

Potencies and efficacies of the examined β_2_AR agonists in the O_2_^•−^ and the cAMP assay are listed in Table 1 and concentration-response curves are depicted in Fig. 3. Additionally, the EC_50_ and *E*_max_ values of ligands determined in steady-state GTPase activity assays using membrane preparations of Sf9 insect cells expressing the β_2_AR-G_sαS_ fusion protein [43], [45] are listed in Table 1. The β_2_AR-G_sαS_ fusion protein is artificial but has become a standard system for the pharmacological analysis of the β_2_AR [43], [45]. For a detailed discussion on the advantages and disadvantages of the fusion protein technique as well as additional references relevant for this approach, the reader is referred to ref. 43. Potencies of ISO, ADR and SAL were higher in the O_2_^•−^ assay than in the cAMP assay. EPH and DCI were lacking agonistic activity in the cAMP assay at concentrations up to 100 µM, whereas inhibitory effects of both ligands on fMLP-stimulated O_2_^•−^ production were readily observed. The efficacy of ADR was comparable in both test systems, but the efficacy of SAL more than two times higher in the O_2_^•−^ assay relative to the cAMP assay. When the data from the recombinant test system were included in the comparison, the rank order of potency of ligands was cAMP assay<GTPase assay<O_2_^•−^ assay, and the rank order of efficacy was cAMP assay<O_2_^•−^ assay ≈ GTPase assay (Fig. 4).

In case of the two-state model postulating a single active state, we would have expected linear correlations for agonists with respect to efficacies and potencies, regardless of which parameters are compared. However, Fig. 4 shows that the correlations are, in general, rather poor, regardless of which comparisons are being made. The worst correlations in terms of deviation from the theoretically expected slope of 1.00 in case of identity of parameters were observed for the comparison of pEC_50_ values in the GTPase and O_2_^•−^ assay (Fig. 4B) and pEC_50_ values in the GTPase and cAMP assay (Fig. 4C). A limitation of our study is that we studied only a limited number of agonists, but an advantage is that the ligands cover a broad range of efficacies and potencies so that clustering of the data in one spot is avoided. In fact, this type of two-dimensional comparison of ligand potencies and efficacies has been repeatedly used to support the concept of ligand-specific receptor conformations in various test systems [24], [27], [32], [40].

A trivial explanation for the differing effects of β_2_AR agonists in the O_2_^•−^ assay and cAMP assay could be that the agonists exhibit O_2_^•−^ scavenging properties on fMLP-stimulated O_2_^•−^ production. However, when O_2_^•−^ production in neutrophils was triggered with phorbol-12-myristate-13-acetate (activator of protein kinase C, 100 nM) instead of fMLP, the examined β_2_AR agonists had no effect on O_2_^•−^ production at all (data not shown).

### Characterization of the β_2_AR on Human Neutrophils with β_2_AR Antagonists

According to conventional models of GPCR activation, potency of an antagonist for a given receptor is constant irrespective of the tissue or recombinant system selected for the characterization, the agonist used for the stimulation of GPCR and downstream signaling event monitored [20]–[22], [47]. However, by monitoring the cAMP accumulation and cAMP response element-mediated reporter gene transcription in Chinese hamster ovary (CHO) cells, different *K*_B_ values were determined for β_2_AR antagonists [48]. These data indicate that antagonists, like agonists, may stabilize functionally distinct receptor conformations. Likewise, we obtained evidence for functional selectivity of antagonists at various recombinant histamine receptor subtypes [49]. Hence, the question arose whether parameter-dependent β_2_AR antagonist potency is also apparent in a native test system, namely in neutrophils.

**Figure 5:**
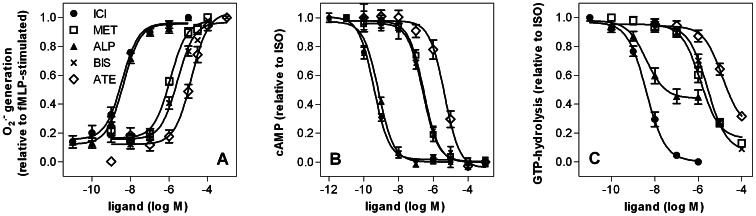
Concentration-response curves for β_2_AR antagonists determined in the O_2_^•^^−^ (A), cAMP (B) and GTPase assay (C). The O_2_^•−^ assay (1×10^5^ cells per well) and the cAMP assay (5×10^5^ cells per cup) were performed as described in sections 2.3 and 2.4, respectively. Steady-state GTPase activity assay, using membrane preparations of Sf9 insect cells, expressing fusion protein β_2_AR-G_sαS_, was performed as described in [43]. Data were analyzed by non-linear regression and were best fitted to sigmoidal concentration/response curves. Data shown are the means ± S.E.M. of four to five independent experiments performed in triplicate. O_2_^•−^ and cAMP production as well as GTP hydrolysis were determined at submaximally effective concentration of ISO (100 nM in the O_2_^•−^ and cAMP assay, 10 nM in the GTPase assay) in the presence of increasing concentrations of β_2_AR antagonists.

**Figure 6:**
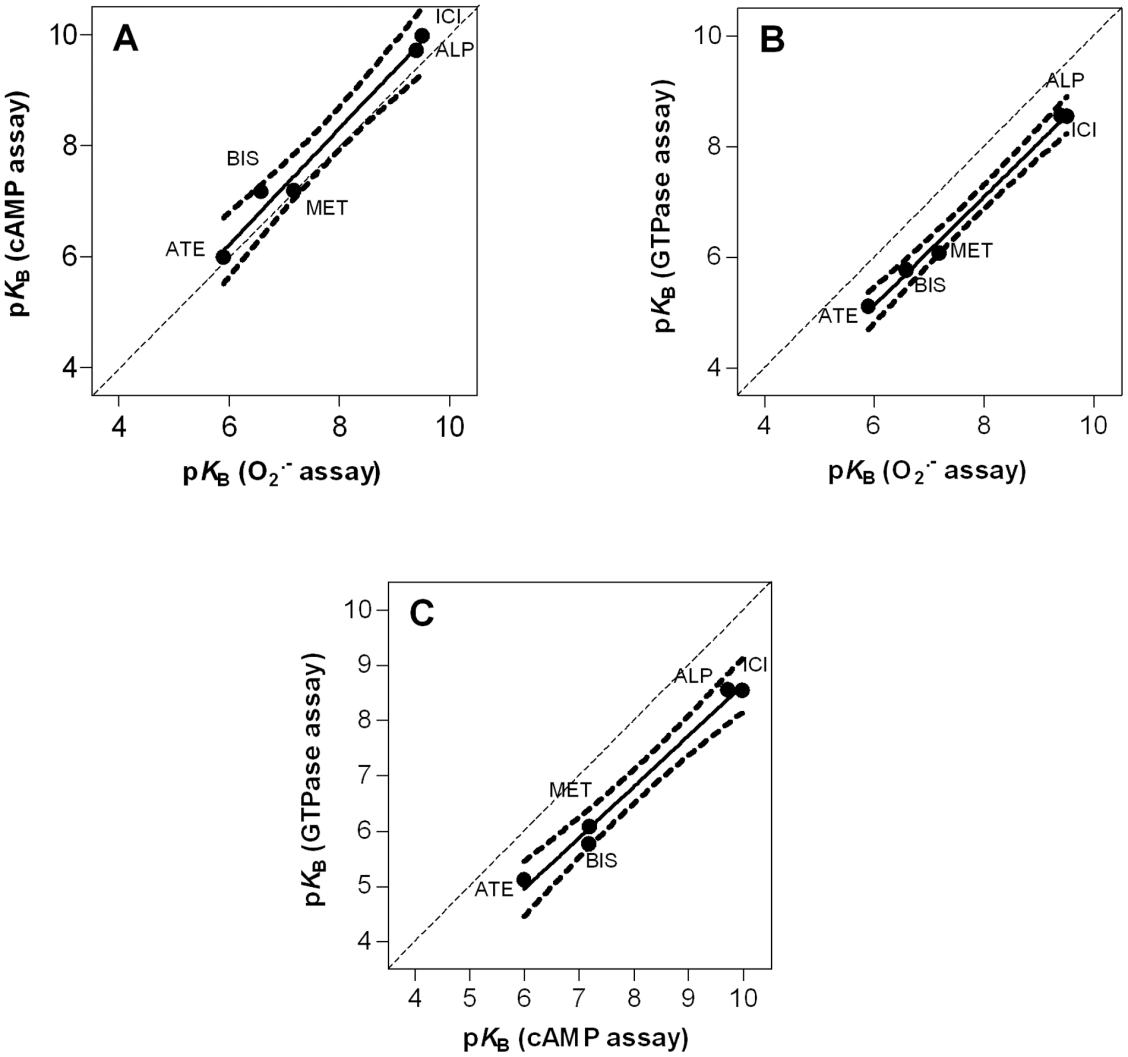
Pair-wise comparison of the p*K*B values of the β2AR antagonists in the O_2_^•^^−^ assay, the cAMP assay and the GTPase assay. The data for comparison were taken from Table 2 and were analyzed by linear regression. The dashed lines represent the 95% confidence intervals in the regression line. The diagonal dotted line indicates a theoretical line for identical values (slope = 1). Slopes (95% confidence interval) and r^2^ of the calculated correlations are as follows; **A.** 1.05 (0.79 to 1.30), 0.98; **B.** 0.98 (0.84 to 1.12), 0.99; **C.** 0.93 (0.73 to 1.12), 0.99.

The p*K*_B_ values for ICI, MET, ALP, BIS and ATE were determined in the cAMP and O_2_^•−^ assay by applying a submaximally effective concentration of ISO and increasing concentrations of β_2_AR antagonists. Concentration-response curves for antagonists are shown in Fig. 5. In Table 2, the results are summarized and compared with antagonistic activity of the same ligands in the recombinant test system (GTPase assay using membranes of Sf9 insect cells expressing β_2_AR-G_sαS_). The statistical analysis of the obtained data revealed no difference of p*K*_B_ values between the two different parameters in neutrophils, BIS being an exception. However, in comparison with data on neutrophils, the potencies of all antagonists were significantly reduced on the recombinantly expressed β_2_AR-G_sαS_. Fig. 6 shows correlations of the p*K*_B_ values of antagonists for the various parameters analyzed. It is evident that the correlations for antagonists are much better than the corresponding correlations for agonists shown in Fig. 5.

**Table 2:** Comparison of p*K*_B_ values of the β_2_AR antagonists, determined in three different test systems.

Cpd.	O_2_^•−^ assay (β_2_AR on neutrophil granulocytes)	cAMP assay (β_2_AR on neutrophil granulocytes)	GTPase assay (recombinant protein β_2_AR-G_sαS_)^a^
	p*K*_B_ *±* S.E.M. *(K_B_ in µM)*	p*K*_B_ *±* S.E.M. *(K_B_ in µM)*	p*K*_B_ *±* S.E.M. *(K_B_ in µM)*
**ICI**	9.51*±*0.09 *(0.00031)*	9.97*±*0.07 *(0.00011)*	8.55*±*0.14***^,+++^ *(0.0028)*
**MET**	7.18*±*0.15 *(0.066)*	7.19*±*0.12 *(0.065)*	6.08*±*0.17**^,++^ *(0.83)*
**ALP**	9.40*±*0.10 *(0.00040)*	9.71*±*0.06 *(0.00020)*	8.56*±*0.14***^,+++^ *(0.0028)*
**BIS**	6.58*±*0.19 *(0.26)*	7.17*±*0.11* *(0.068)*	5.77*±*0.10**^,+++^ *(1.70)*
**ATE**	5.89*±*0.12 *(1.29)*	5.99*±*0.20 *(1.02)*	5.12*±*0.17*^,+^ *(7.59)*

On human neutrophil granulocytes, the O_2_^•−^ assay (1×10^5^ cells per well) and the cAMP assay (5×10^5^ cells per sample) were performed as described under *Materials and Methods*. Steady-state GTPase activity assay, using membrane preparations of Sf9 insect cells, expressing fusion protein β_2_AR-G_sαS_, was performed as described in [43]. O_2_^•−^ and cAMP production as well as GTP hydrolysis were determined at submaximally effective concentration of ISO (100 nM in the O_2_^•−^ and cAMP assay, 10 nM in the GTPase assay) in the presence of increasing concentrations of β_2_AR antagonists. Data were analyzed by non-linear regression and were best fitted to sigmoidal concentration/response curves. Data shown are from four to five independent experiments performed in triplicate. The p*K*_B_ values were calculated from the IC_50_ values according to Cheng and Prusoff [44]. p*K*_B_ values were compared with each other using one-way ANOVA, followed by Bonferroni’s multiple comparison test (p*K*_B_ significantly different to: *O_2_^•−^ assay, ^+^cAMP assay; one symbol: p<0.05, two symbols: p<0.01, three symbols: p<0.001). Non-logarithmic *K*_B_ values in µM are shown in parentheses.

### Do AC- and PKA-activation Interfere with fMLP-stimulated O_2_^•−^ Production?

**Figure 7:**
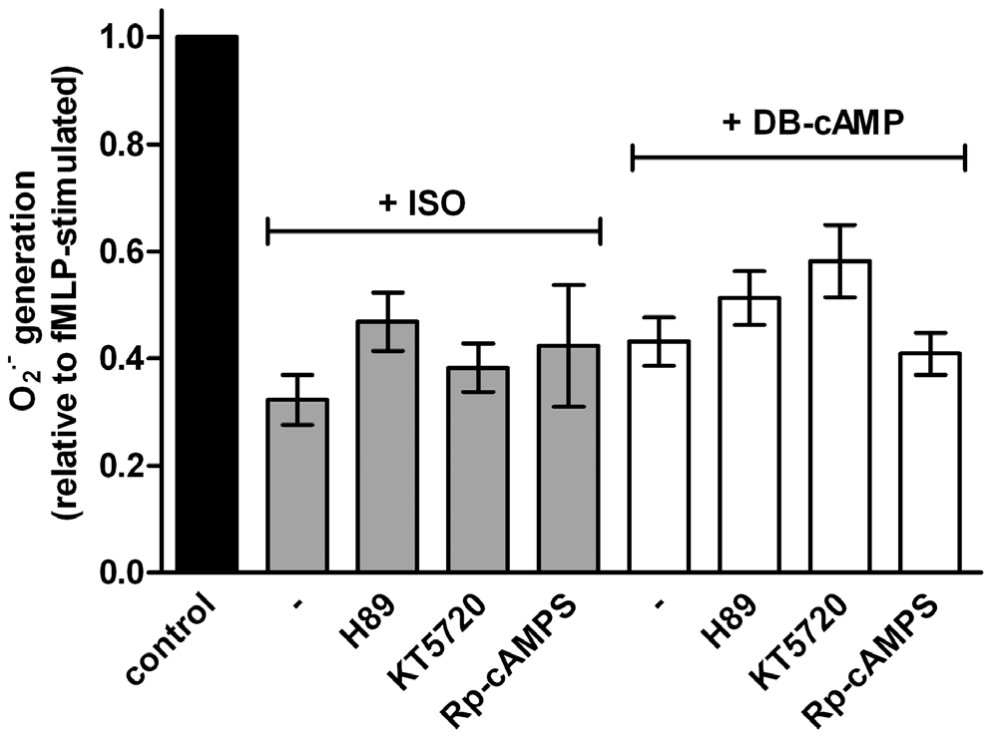
Effects of different PKA inhibitors on the ISO- and DB-cAMP-induced inhibition of fMLP-stimulated O_2_^•^^−^ production. O_2_^•−^ production in human neutrophil granulocytes (1×10^5^ cells per well) was monitored by measuring the superoxide dismutase-inhibitable reduction of ferricytochrome c at 550 nm. Data shown (mean ± S.E.M.) are from three independent experiments, performed in triplicate. Concentrations used are as follows; ISO 100 nM, DB-cAMP 500 µM, H89 1 µM, KT5720 10 µM and Rp-cAMPS 100 µM. H89, KT5720 and Rp-cAMPS had no significant reversal effect on the inhibitions caused by ISO and DB-cAMP.

As already mentioned in the introduction, β_2_AR-signaling is very complex, depending on ligand and test system [15], [16], [18], [26], [27]. β_2_AR-mediated inhibition of fMLP-stimulated O_2_^•−^ production is thought to be due to cAMP production and PKA activation [50]–[51]. In order to address this question we studied the effects of three structurally distinct and well-established PKA inhibitors. Among the inhibitors are an isoquinoline sulfonamide [52], a cAMP analog [53] and a microbial metabolite [54]. However, in our experiments, we failed to obtain evidence for the hypothesis that activation of PKA after β_2_AR stimulation is a crucial event for inhibition of fMLP-stimulated O_2_^•−^ production (Fig. 7). Specifically, compounds H89 and KT5720, widely-used and effective cell-permeable competitive inhibitors of the ATP-binding to the ATP-binding pocket of the PKA in other test systems [55]–[58], did not reverse ISO-induced inhibition of fMLP-stimulated O_2_^•−^ production (also when neutrophils were pretreated 15 min with H89 or KT5720). Even the cAMP antagonist Rp-cAMPS which competes with cAMP for the binding to the regulatory subunits of PKA [57], [59], did not interfere with the effect of ISO of O_2_^•−^ production in human neutrophils (Fig. 7).

**Figure 8:**
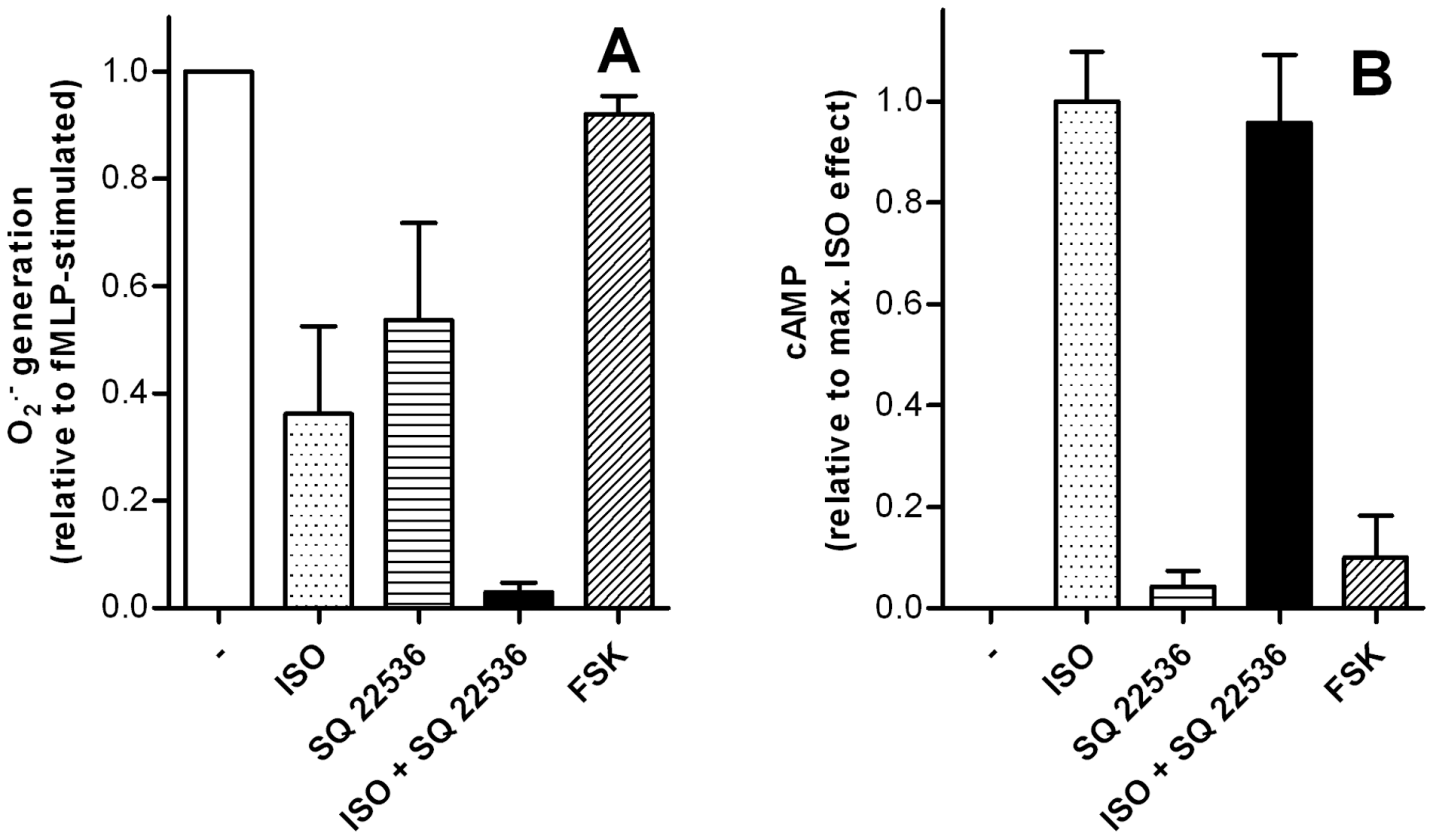
Effects of SQ 22536 and FSK on the fMLP-stimulated O_2_^•^^−^ production (A) and cAMP accumulation (B). The O_2_^•−^ assay (1×10^5^ cells per well) and the cAMP assay (5×10^5^ cells per cup) were performed as described in Materials and Methods. Data shown (mean ± S.E.M.) are from three to six independent experiments, performed in triplicate. Concentrations used are as follows; ISO 100 nM (**A**) and 1 µM (**B**), SQ 22536 100 µM. For comparison, we also studied the effect of the direct AC activator FSK (10 µM). In all tubes, a final concentration of 1% (v/v) DMSO (unavoidable for dissolving SQ 22536 and FSK) was present to achieve comparable results. SQ 22536 had no significant reversal effect on the inhibition caused by ISO in the O_2_^•−^ assay, and FSK had no significant inhibitory effect on fMLP stimulation in this assay.

Moreover, we tried to assess the involvement of the cAMP signaling pathway in the fMLP-stimulated O_2_^•−^ production by applying the AC inhibitor SQ 22536 [60]. Surprisingly, SQ 22536 enhanced rather than diminished the inhibitory effect of ISO on fMLP-induced O_2_^•−^ production (Fig. 8A). Additionally, SQ 22536 exhibited unexpected inhibitory effects on fMLP-induced O_2_^•−^ production on its own. SQ 22536 by itself did not increase cAMP levels in neutrophils, and the compound also did not inhibit the ISO-induced cAMP increase (Fig. 8B). Pleiotropic and AC-independent effects of SQ 22536 have been observed repeatedly [61].

As an additional tool we examined the diterpene, FSK, a direct activator of membranous ACs [61]. However, FSK did neither significantly reduce fMLP-stimulated O_2_^•-^ production (Fig. 8A) nor robustly increase cAMP levels (Fig. 8B).

**Figure 9:**
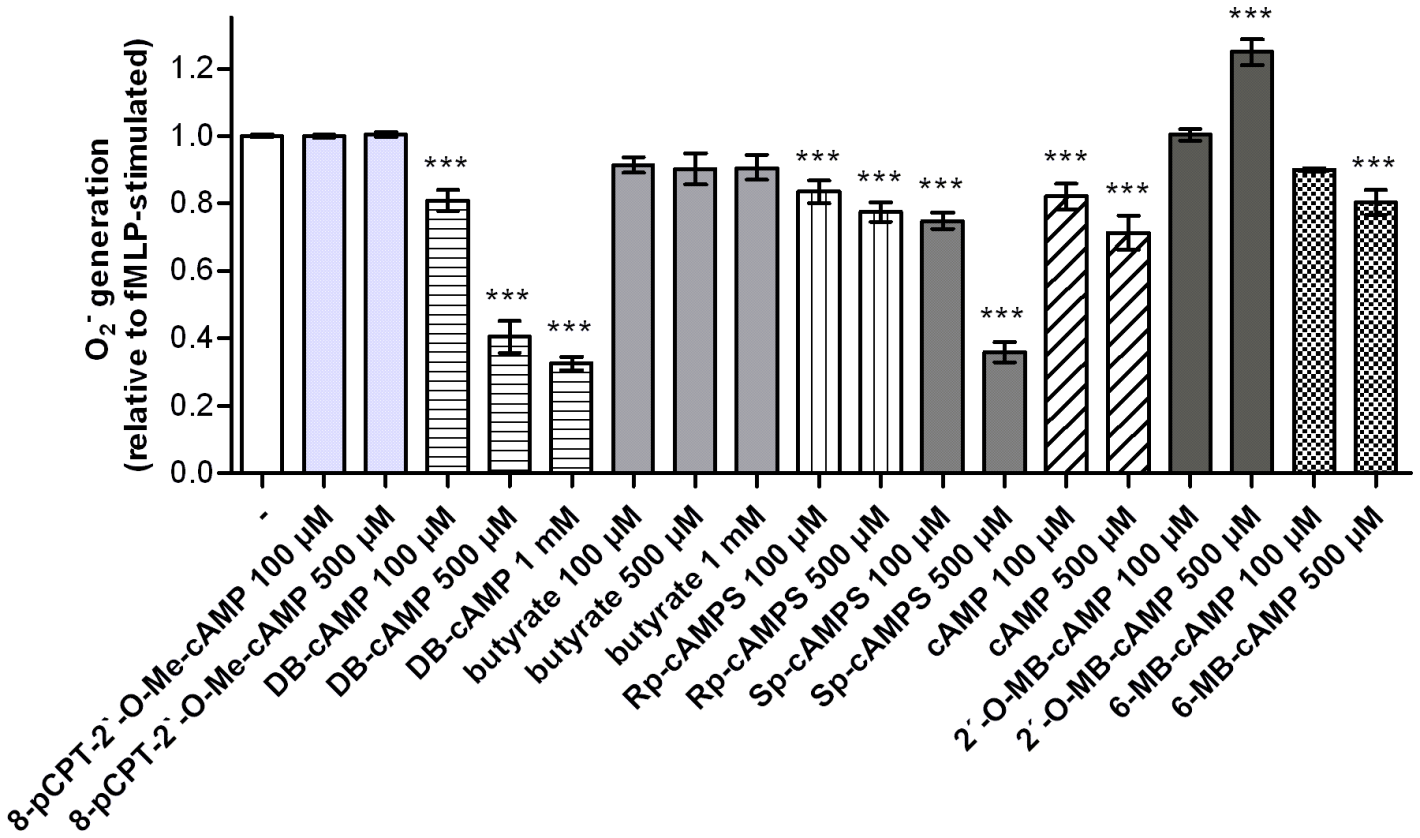
Effects of cAMP, butyrate and cAMP analogs on fMLP-stimulated O_2_^•^^−^ production. O_2_^•−^ production in human neutrophil granulocytes (1×10^5^ cells per well) was monitored by measuring superoxide dismutase-inhibitable reduction of ferricytochrome c at 550 nm as described in *Materials and Methods*. Data shown (mean ± S.E.M.) are from three to seven independent experiments, performed in triplicate. Data were analyzed for statistical significance relative to the control (-, set to 1.0) using one-way ANOVA, followed by Dunnett’s multiple comparison test (***p<0.001).

In order to provide proof of principle that an increase in intracellular cAMP concentration is capable of inhibiting fMLP-stimulated O_2_^•−^ production, we examined the effects of cAMP and various cAMP analogs on NADPH oxidase activation. DB-cAMP is lipophilic and penetrates the plasma membrane. Inside the cell, DB-cAMP is converted to the biologically active 6-MB-cAMP [62]. In accordance with previous data [9], DB-cAMP reduced fMLP-stimulated O_2_^•−^ production, whereas the control compound sodium butyrate was ineffective (Fig. 9). In addition, the mono-butyrylated control compound 6-MB-cAMP did not robustly inhibit NADPH oxidase, most likely due to inefficient membrane penetration. Sp-cAMPS is less lipophilic than DB-cAMP but does not require bioactivation [59]. Sp-cAMPS was similarly effective at inhibiting O_2_^•−^ production as DB-cAMP. These data show that cAMP does have the potential to inhibit NADPH oxidase. However, we also noted that very high concentrations of DB-cAMP are required to elicit inhibition, probably exceeding the intracellular cAMP concentrations achieved following β_2_AR stimulation.

cAMP itself also slightly inhibited O_2_^•−^ production. This could be due to extracellular degradation of cAMP to adenosine by phosphodiesterases and ectonucleotidases and subsequent activation of adenosine A2-receptors by adenosine [63]. Import of cAMP via multidrug resistance protein transporters (MRPs) into cells and subsequent PKA activation could be involved as well [64]. Likewise, the small inhibitory effects of the PKA inhibitor Rp-cAMPS could be due to adenosine liberation from the parent compound. We do not have a satisfactory explanation for the small but significant stimulatory effect of the mono-butyrylated control compound 2′-O-MB-cAMP on O_2_^•−^ production. However, we confirmed that the compound per se did not activate O_2_^•−^ production or reduced ferricytochrome c independently of NADPH oxidase (data not shown). The activator of the cAMP effector protein Epac, 8-pCPT-2′-O-Me-cAMP [65], did not inhibit fMLP-induced O_2_^•−^ production, arguing against an involvement of Epac in NADPH oxidase regulation. We also observed that the PKA inhibitors H89, KT5720 and Rp-cAMPS showed no reversing effect on the inhibition of O_2_^•−^ production caused by DB-cAMP (Fig. 7). These data raise questions whether a hitherto unidentified cAMP-binding protein is involved in the inhibition of O_2_^•−^ production by DB-cAMP and Sp-cAMPS.

## Discussion

The two-state model of receptor activation implying an active (R*) and an inactive (R) state has now been superseded by a more complex model involving multiple active receptor conformations that lead to ligand-specific receptor activation, also referred to as functional selectivity or biased agonism [19]–[33]. Functional selectivity has been reported for numerous GPCRs such as dopamine D_1_ and D_2_ receptors, the histamine H_2_ and H_4_ receptor, adenosine A_1_ and A_3_ receptors, the α_2A_-adrenoceptor and the β_2_AR [15]–[33], [40], [49]. So-called biased ligands can differently activate G protein-dependent and -independent signaling such as the β-arrestin pathway [27], [32], [34], [35], can discriminate between G_s_, G_i_, G_q_ and other G protein-mediated pathways [15], [66] or even selectively modulate e.g. G_i1_, G_i2_ and G_i3_ protein subtype activities [67]. Therefore, it is not surprising that any given ligand possesses multiple potencies and efficacies depending on the down-stream pathway analyzed [33]. This concept was also confirmed in our study with β_2_AR agonists in human neutrophils using the cAMP assay and the O_2_^•−^ assay as parameters and by comparison of the results with literature data obtained in recombinant test system (Fig. 4). If only a single active β_2_AR conformation existed, we would have expected linear correlations following the dotted lines in Fig. 4 between potencies and efficacies (relative to the reference compound ISO) of agonists, regardless of which parameters are considered. However, this was not the case. In accordance with our data, fluorescence studies with purified β_2_AR nuclear magnetic resonance studies provided evidence for ligand-specific conformations [31], [37], [39].

With respect to β_2_AR antagonists, effects were similar in the cAMP and O_2_^•−^ assay on neutrophils (Table 2 and Fig. 5), indicating that in neutrophils, functional selectivity is predominantly observed for β_2_AR agonists. In contrast to neutrophil parameters, potencies of antagonists were generally lower at the recombinant β_2_AR than at the native β_2_AR (Table 2), and there were also ligand-specific differences. The trend towards lower antagonist (inverse agonist) potencies at the recombinant β_2_AR could be due to higher constitutive activity of the recombinant than of the native system [20].

The vast majority of reports about functional selectivity originate from studies with recombinant test systems or purified receptors (see, e.g., [31], [34]–[36], [38]–[39]). On the contrary, functional selectivity in native test systems has been rarely studied so far, e.g. for the histamine H_4_ receptor on isolated human eosinophils [68] and for the histamine H_2_ receptor on isolated human eosinophils and neutrophils [40]. Here, we report on functional selectivity of the β_2_AR in human neutrophils. In accordance with our data, there is evidence for functional selectivity of β_2_AR ligands in cardiomyocytes [69]. In this system, stereoisomers of fenoterol differentially activate G_i_- and G_s_-proteins.

Unfortunately, in-depth analysis of functional selectivity in neutrophils is hampered by limited possibilities to block coupling of the β_2_AR to coupling partners. GPCR-G_i_ protein coupling can be interrupted with pertussis toxin [70], whereas there is no pharmacological tool available for the selective inhibition of G_s_ and G_q_ coupling or the β-arrestin pathway in native test systems. Furthermore, since the formyl peptide receptor is coupled to G_i_-proteins and an essential stimulatory component in the O_2_^•−^ assay, we could not use pertussis toxin to differentiate between β_2_AR ligands in this assay and in the cAMP assay. Moreover, in neutrophils, difficulties for effective genetic manipulation, inter-individual variability and relatively short life time impede with more detailed analysis of functional selectivity in this native test system.

Our data obtained by measuring GTP hydrolysis in the recombinant test system reflect coupling of the β_2_AR solely to the short splice variant of the G_s_ protein [43], [45]. There is no doubt that the β_2_AR couples to G_s_ in neutrophils, but no information is available regarding the involvement of specific G_s_ splice variants. The question remains what the reason for the generally increased potency of the examined β_2_AR agonists in the O_2_^•−^ assay compared to the cAMP assay is. Lack of correlation between the cAMP accumulation and inhibition of fMLP-induced O_2_^•−^ production in neutrophils has been described also by other research groups using other stimuli [71]–[74]. Among other reasons, better coupling efficiency of the β_2_AR to the O_2_^•−^ pathway than to the cAMP pathway is of relevance for the divergence in data. Differences in strength of coupling to different signaling pathways have been reported for other receptors [75]. Additionally, we have no evidence for the involvement of Epac in the signal transduction pathway of the β_2_AR leading to inhibition of NADPH oxidase since an effective Epac activator failed to inhibit fMLP-stimulated O_2_^•−^ production (Fig. 9).

We failed to support an involvement of AC, cAMP and PKA in β_2_AR-mediated inhibition of fMLP-stimulated O_2_^•−^ production. Thus, it appears that the two measured events (cAMP accumulation and O_2_^•−^ production) in neutrophils are independent of each other. The lack of effect of DCI and EPH on cAMP accumulation despite inhibitory effects of these ligands on O_2_^•−^ production supports the concept of cAMP-independent inhibition of NADPH oxidase. Moreover, SAL is more effective at inhibiting NADPH oxidase than at increasing cAMP. Our failure to detect stimulatory effects of DCI and EPH on cAMP accumulation and ineffective stimulation of cAMP accumulation by SAL are not due to cAMP degradation since we included a phosphodiesterase inhibitor into the cAMP assay. Moreover, we applied a highly sensitive and specific MS method to detect cAMP, avoiding notorious cross-reactivity problems of antibodies widely applied in cyclic nucleotide detection [41], [42], [76]. Thus, our study addressing ligand-specific receptor conformations also casted doubt about the dogma of cAMP-dependent inhibition of NADPH oxidase by the β_2_AR although, in principle, cAMP can inhibit O_2_^•−^ production (Fig. 9). Even in case of inhibition of O_2_^•−^ production by cAMP analogs, we failed to obtain positive evidence for an involvement of PKA (Fig. 7).

Analysis of the signaling pathways responsible for inhibition of O_2_^•−^ production in neutrophils is hampered by unsuitability of experimental tools available. Most strikingly, the widely used AC inhibitor SQ 22536 failed to reduce the stimulatory effect of ISO on cAMP levels but further augmented the inhibitory effect of ISO on O_2_^•−^ production (Fig. 8). Non-specific and pleiotropic effects of SQ 22536 have been subject of a recent review [61]. Quite striking too was the lack of inhibitory effect of FSK on O_2_^•−^ production and lack of stimulatory effect of FSK on cAMP production (Fig. 8). These data could be explained by a model according to which the FSK-insensitive AC isoform 9 [61] is the functionally predominant AC in neutrophils. This hypothesis needs to be tested in future studies. Again, this is not a trivial task since the quality of AC antibodies is generally poor [61], and we are not aware of the availability of specific AC9 antibodies.

Stimulation of cAMP accumulation and reduction of O_2_^•−^ production mediated by ISO, was inhibited according to monophasic competition isotherms by ICI, a highly potent and selective β_2_AR antagonist with very low potency on the β_1_-adrenergic receptor [77]. In case of an exclusive involvement of the β_1_-adrenergic receptor we would have expected low potency of ICI, and in case of an involvement of both β-adrenergic receptors, we would have expected biphasic isotherms. This was clearly not the case (Fig. 5), and moreover, the potency of ICI at the native β_2_AR was even higher than at the recombinant β_2_AR (Fig. 5 and Table 2). Thus, the data obtained with ICI provide strong evidence for the notion that only the β_2_AR but not the β_1_-adrenergic receptor is functionally expressed in human neutrophils. Moreover, we excluded the possibility that ISO as representative β_2_AR agonist cross-reacts with the histamine H_2_ receptor, which is also expressed on human neutrophils [40]. Specifically, the effect of ISO on fMLP-induced O_2_^•−^ production was not reduced by the histamine H_2_ receptor antagonists famotidine, tiotidine and zolantidine (data not shown).

Stallaert and coworkers [78] demonstrated on HEK293S cells that β_2_AR-dependent impedance response to ISO is the result of activation of multiple signaling pathways, including G_s_ and G_i_ coupling, Gβγ-dependent signaling, cAMP production, extracellular signal-regulated kinase (ERK) 1/2 activation as well as Ca^2+^ mobilization. Therefore, when stimulation of the β_2_AR does not activate the PKA-dependent pathway in human neutrophils or this pathway does not interfere with the NADPH oxidase signaling, modulation of the e.g. ERK1/2-pathway could be the explanation for the inhibition of the NADPH oxidase. Interestingly, in other studies on human neutrophils, a correlation between activation of cAMP/PKA signaling pathway and inhibition of ERK phosphorylation was observed, resulting in reduced fMLP-induced O_2_^•−^ production [79], [80]. Intriguingly, the β_2_AR was reported to activate ERK signaling pathway *via* interaction with G_i_, Src and/or arrestin proteins in addition to G_s_ proteins in other systems [81]. As it is evident that β_2_AR-signalling strongly depends on the cell system used [16], there is a need to address the correlation between β_2_AR activation, ERK activation and fMLP-stimulated O_2_^•−^ production in future studies as well. This could provide an explanation for the observed biased effects of some examined β_2_AR ligands on neutrophils.

In preliminary studies we examined a number of pharmacological inhibitors to explore alternative signaling pathways of the β_2_AR; e.g. we tested the p38 inhibitor SB203580, the JNK inhibitors SP600125 and SP600123, the ERK inhibitor PD980598, the PI3 kinase inhibitor LY294002 and the protein kinase C inhibitor Goe 6978. Unfortunately, these compounds per se inhibited fMLP-stimulated O_2_^•−^ production (data not shown) so that separate effects of these compounds on ISO actions could not be properly dissected. An alternative approach will be the examination of the effects of β_2_AR ligands on protein phosphorylation in neutrophils, using screening approaches encompassing numerous protein kinases as starting point. Lastly, it will also be very important to explore the possibility that the new signaling molecules cyclic CMP (cCMP) and cyclic UMP (cUMP) [82] are involved in β_2_AR-mediated signal transduction.

Data from the literature suggest a feasible use of β_2_AR agonists as anti-inflammatory agents [10], [83]. In order to extend the data on functional selectivity in native test systems, we compared the effect of β_2_AR ligands on cAMP accumulation and O_2_^•−^ production in human neutrophils. However, there are many native test systems described, which would afford an expansion of our knowledge about functional selectivity of β_2_AR ligands. One example could be the parallel measurement of cAMP concentration and contractility of cardiomyocytes or endothelial cells as functional parameter [16], [69]. Moreover, a comparison of cAMP accumulation and ERK phosphorylation in e.g. mouse embryonic fibroblasts [84] could be used for screening of β_2_AR ligands. All in all, there is a need to assess biased signaling through the β_2_AR in a wide spectrum of native test systems in order to improve the desired therapeutic effect of developed compounds on the one and to minimize side effects on the other. Furthermore, β_2_AR agonists that have been used for many years in the therapy of humans, e.g. as bronchodilators in patients with asthma or chronic obstructive pulmonary disease, should be reassessed using various native test systems, as there is a potential to improve already existing therapies, particularly by minimizing unwanted effects.

As a general approach to study functional selectivity in native cells, it is necessary to construct a systematic data matrix in which multiple ligands are examined at multiple concentrations (enabling determination of precise potencies and efficacies) and for multiple parameters. Previous studies with native human cells may have largely overlooked functional selectivity at GPCRs because there was no appreciation of the necessity to generate such a systematic data collection in order to understand cell biology. It is evident that availability of human cells is an issue for comprehensive pharmacological studies. The human neutrophil, despite its inherent problems, i.e. short survival time after isolation, variable responsiveness and poor accessibility to genetic manipulation, is a suitable model system to test functional selectivity for several reasons. Specifically, neutrophils can be obtained in large quantities, express multiple receptors, display numerous cell functions that can be assessed quantitatively and are pathophysiologically relevant for inflammation.

## Supporting Information

Figure S1 **Structures of the β_2_AR agonists and antagonists examined in this study.**
(PPTX)

Figure S2 **Structures of cAMP and cAMP analogs examined in this study.**
(PPTX)

## References

[1] Selvatici R, Falzarano S, Mollica A, Spisani S (2006) Signal transduction pathways triggered by selective formylpeptide analogues in human neutrophils. Eur J Pharmacol 534: 1–11. 16516193 10.1016/j.ejphar.2006.01.034

[2] Seifert R, Schultz G (1991) The superoxide-forming NADPH oxidase of phagocytes. An enzyme system regulated by multiple mechanisms. Rev Physiol Biochem Pharmacol 117: 1–338. 1659735

[3] Morel F, Doussiere J, Vignais PV (1991) The superoxide-generating oxidase of phagocytic cells. Physiological, molecular and pathological aspects. Eur J Biochem 201: 523–546. 1657601 10.1111/j.1432-1033.1991.tb16312.x

[4] El-Benna J, Dang PM, Perianin A (2010) Peptide-based inhibitors of the phagocyte NADPH oxidase. Biochem Pharmacol 80: 778–785. 20510204 10.1016/j.bcp.2010.05.020

[5] Arruda MA, Barja-Fidalgo C (2009) NADPH oxidase activity: In the crossroad of neutrophil life and death. Front Biosci 14: 4546–4556. 10.2741/3547 19273369

[6] Burde R, Seifert R, Buschauer A, Schultz G (1989) Histamine inhibits activation of human neutrophils and HL-60 leukemic cells *via* H_2_ receptors. Naunyn-Schmiedeberg's Arch Pharmacol 340: 671–678. 2559336 10.1007/BF00717743

[7] Gierschik P, Sidiropoulos D, Jakobs KH (1989) Two distinct G_i_ proteins mediate formyl peptide receptor signal transduction in human leukemia (HL-60) cells. J Biol Chem 264: 21470–21473. 2513319

[8] Wenzel-Seifert K, Arthur JM, Liu HY, Seifert R (1999) Quantitative analysis of formyl peptide receptor coupling to Gα_i1_, Gα_i2_, and Gα_i3_ . J Biol Chem 274: 33259–33266. 10559200 10.1074/jbc.274.47.33259

[9] Wenzel-Seifert K, Ervens J, Seifert R (1991) Differential inhibition and potentiation by cell-permeant analogues of cyclic AMP and cyclic GMP and NO-containing compounds of exocytosis in human neutrophils. Naunyn Schmiedebergs Arch Pharmacol 344: 396–402. 1722562 10.1007/BF00172578

[10] Mirza ZN, Kato M, Kimura H, Tachibana A, Fujiu T, et al (2002) Fenoterol inhibits superoxide anion generation by human polymorphonuclear leukocytes via β-adrenoceptor-dependent and -independent mechanisms. Ann Allergy Asthma Immunol 88: 494–500. 12027071 10.1016/s1081-1206(10)62388-5

[11] Mitsuyama T, Takeshige K, Furuno T, Tanaka T, Hidaka K, et al (1995) An inhibitor of cyclic AMP-dependent protein kinase enhances the superoxide production of human neutrophils stimulated by N-formyl-methionyl-leucyl-phenylalanine. Mol Cell Biochem 145: 19–24. 7659074 10.1007/BF00925708

[12] Mueller H, Motulsky HJ, Sklar LA (1988) The potency and kinetics of the β-adrenergic receptors on human neutrophils. Mol Pharmacol 34: 347–353. 2901665

[13] Wong K, Freund K (1981) Inhibition of the N-formylmethionyl-leucyl-phenylalanine induced respiratory burst in human neutrophils by adrenergic agonists and prostaglandins of the E series. Can J Physiol Pharmacol 59: 915–920. 6271378 10.1139/y81-141

[14] Lad PM, Goldberg BJ, Smiley PA, Olson CV (1985) Receptor-specific threshold effects of cyclic AMP are involved in the regulation of enzyme release and superoxide production from human neutrophils. Biochim Biophys Acta 846: 286–295. 2411298 10.1016/0167-4889(85)90076-x

[15] Wenzel-Seifert K, Seifert R (2000) Molecular analysis of β_2_-adrenoceptor coupling to G_s_, G_i_, and G_q_ proteins. Mol Pharmacol 58: 954–966. 11040042 10.1124/mol.58.5.954

[16] Evans BA, Sato M, Sarwar M, Hutchinson DS, Summers RJ (2010) Ligand-directed signalling at β-adrenoceptors. Br J Pharmacol 159: 1022–1038. 20132209 10.1111/j.1476-5381.2009.00602.x PMC2839261

[17] Drake MT, Violin JD, Whalen EJ, Wisler JW, Shenoy SK, et al (2008) β-Arrestin-biased agonism at the β_2_-adrenergic receptor. J Biol Chem 283: 5669–5676. 18086673 10.1074/jbc.M708118200

[18] Audet M, Bouvier M (2008) Insights into signaling from the β_2_-adrenergic receptor structure. Nat Chem Biol 4: 397–403. 18560432 10.1038/nchembio.97

[19] Rosenbaum DM, Rasmussen SG, Kobilka BK (2009) The structure and function of G-protein-coupled receptors. Nature 459: 356–363. 19458711 10.1038/nature08144 PMC3967846

[20] Seifert R, Wenzel-Seifert K (2002) Constitutive activity of G protein-coupled receptors: cause of disease and common property of wild-type receptors. Naunyn Schmiedebergs Arch Pharmacol 366: 381–416. 12382069 10.1007/s00210-002-0588-0

[21] Neubig RR, Spedding M, Kenakin T, Christopoulos A (2003) International Union of Pharmacology Committee on Receptor Nomenclature and Drug Classification. XXXVIII. Update on terms and symbols in quantitative pharmacology. Pharmacol Rev 55: 597–606. 14657418 10.1124/pr.55.4.4

[22] Kenakin T (2004) Principles: receptor theory in pharmacology. Trends Pharmacol Sci 25: 186–192. 15063082 10.1016/j.tips.2004.02.012

[23] Gether U, Lin S, Kobilka BK (1995) Fluorescent labeling of purified β_2_-adrenergic receptor. Evidence for ligand-specific conformational changes. J Biol Chem 270: 28268–28275. 7499324 10.1074/jbc.270.47.28268

[24] Seifert R, Gether U, Wenzel-Seifert K, Kobilka BK (1999) Effects of guanine, inosine, and xanthine nucleotides on β_2_-adrenergic receptor/G_s_ interactions: evidence for multiple receptor conformations. Mol Pharmacol 56: 348–358. 10419554 10.1124/mol.56.2.348

[25] Sternini C, Spann M, Anton B, Keith DE Jr, Bunnett NW, et al (1996) Agonist-selective endocytosis of μ opioid receptor by neurons *in vivo* . Proc Natl Acad Sci USA 93: 9241–9246. 8799185 10.1073/pnas.93.17.9241 PMC38626

[26] Galandrin S, Bouvier M (2006) Distinct signaling profiles of β_1_- and β_2_-adrenergic receptor ligands toward adenylyl cyclase and mitogen-activated protein kinase reveals the pluridimensionality of efficacy. Mol Pharmacol 70: 1575–1584. 16901982 10.1124/mol.106.026716

[27] Rajagopal S, Rajagopal K, Lefkowitz RJ (2010) Teaching old receptors new tricks: biasing seven-transmembrane receptors. Nat Rev Drug Discov 9: 373–386. 20431569 10.1038/nrd3024 PMC2902265

[28] Kobilka BK, Deupi X (2007) Conformational complexity of G protein-coupled receptors. Trends Pharmacol Sci 28: 397–406. 17629961 10.1016/j.tips.2007.06.003

[29] Kenakin T (1995) Agonist-receptor efficacy. II. Agonist trafficking of receptor signals. Trends Pharmacol Sci 16: 232–238. 7667897 10.1016/s0165-6147(00)89032-x

[30] Tucek S (1997) Is the R and R dichotomy real? Trends Pharmacol Sci 18: 414–416. 9426467 10.1016/s0165-6147(97)01123-1

[31] Liu JJ, Horst R, Katritch V, Stevens RC, Wüthrich K (2012) Biased signaling pathways in β_2_-adrenergic receptor characterized by 19F-NMR. Science 335: 1106–1110. 22267580 10.1126/science.1215802 PMC3292700

[32] Galandrin S, Oligny-Longpré G, Bouvier M (2007) The evasive nature of drug efficacy: implications for drug discovery. Trends Pharmacol Sci 28: 423–430. 17659355 10.1016/j.tips.2007.06.005

[33] Kenakin T, Miller LJ (2010) Seven transmembrane receptors as shapeshifting proteins: the impact of allosteric modulation and functional selectivity on new drug discovery. Pharmacol Rev 62: 265–304. 20392808 10.1124/pr.108.000992 PMC2879912

[34] Rosethorne EM, Charlton SJ (2011) Agonist-biased signaling at the histamine H_4_ receptor: JNJ7777120 recruits β-arrestin without activating G proteins. Mol Pharmacol 79: 749–757. 21134907 10.1124/mol.110.068395

[35] Azzi M, Charest PG, Angers S, Rousseau G, Kohout T, et al (2003) β-arrestin-mediated activation of MAPK by inverse agonists reveals distinct active conformations for G protein-coupled receptors. Proc Natl Acad Sci USA 100: 11406–11411. 13679574 10.1073/pnas.1936664100 PMC208770

[36] Thomas RL, Mistry R, Langmead CJ, Wood MD, Challiss RA (2008) G protein coupling and signaling pathway activation by M_1_ muscarinic acetylcholine receptor orthosteric and allosteric agonists. J Pharmacol Exp Ther 327: 365–374. 18664591 10.1124/jpet.108.141788

[37] Swaminath G, Deupi X, Lee TW, Zhu W, Thian FS, et al (2005) Probing the β_2_-adrenoceptor binding site with catechol reveals differences in binding and activation by agonists and partial agonists. J Biol Chem 280: 22165–22171. 15817484 10.1074/jbc.M502352200

[38] Kahsai AW, Xiao K, Rajagopal S, Ahn S, Shukla AK, et al (2011) Multiple ligand-specific conformations of the β_2_-adrenergic receptor. Nat Chem Biol 7: 692–700. 21857662 10.1038/nchembio.634 PMC3404607

[39] Granier S, Kim S, Shafer AM, Ratnala VR, Fung JJ, et al (2007) Structure and conformational changes in the C-terminal domain of the β_2_-adrenoceptor: insights from fluorescence resonance energy transfer studies. J Biol Chem 282: 13895–13905. 17347144 10.1074/jbc.M611904200

[40] Reher TM, Brunskole I, Neumann D, Seifert R (2012) Evidence for ligand-specific conformations of the histamine H_2_-receptor in human eosinophils and neutrophils. Biochem Pharmacol 84: 1175–1185. 10.1016/j.bcp.2012.08.014 22922404

[41] Beste KY, Burhenne H, Kaever V, Stasch JP, Seifert R (2012) Nucleotidyl cyclase activity of soluble guanylyl cyclase α_1_β_1_ . Biochemistry 51: 194–204. 22122229 10.1021/bi201259y

[42] Kinast L, von der Ohe J, Burhenne H, Seifert R (2012) Impairment of adenylyl cyclase 2 function and expression in hypoxanthine phosphoribosyltransferase-deficient rat B103 neuroblastoma cells as model for Lesch-Nyhan disease: BODIPY-forskolin as pharmacological tool. Naunyn Schmiedebergs Arch Pharmacol 385: 671–683. 22552731 10.1007/s00210-012-0759-6

[43] Weitl N, Seifert R (2008) Distinct interactions of human β_1_- and β_2_-adrenoceptors with isoproterenol, epinephrine, norepinephrine, and dopamine. J Pharmacol Exp Ther 327: 760–769. 18772317 10.1124/jpet.108.143412

[44] Cheng Y, Prusoff WH (1973) Relationship between the inhibition constant (K1) and the concentration of inhibitor which causes 50 per cent inhibition (I50) of an enzymatic reaction. Biochem Pharmacol 22: 3099–3108. 4202581 10.1016/0006-2952(73)90196-2

[45] Seifert R, Wenzel-Seifert K, Lee TW, Gether U, Sanders-Bush E, et al (1998) Different effects of G_sα_ splice variants on β_2_-adrenoreceptor-mediated signaling. The β_2_-adrenoreceptor coupled to the long splice variant of G_sα_ has properties of a constitutively active receptor. J Biol Chem 273: 5109–5116. 9556548

[46] Seifert R, Hilgenstock G, Fassbender M, Distler A (1991) Regulation of the superoxide-forming NADPH oxidase of human neutrophils is not altered in essential hypertension. J Hypertens 9: 147–153. 1849530 10.1097/00004872-199102000-00008

[47] Hill SJ (2006) G protein-coupled receptors: past, present and future. Br J Pharmacol 147 Suppl 1S27–37. 16402114 10.1038/sj.bjp.0706455 PMC1760739

[48] Baker JG, Hall IP, Hill SJ (2003) Influence of agonist efficacy and receptor phosphorylation on antagonist affinity measurements: differences between second messenger and reporter gene responses. Mol Pharmacol 64: 679–688. 12920204 10.1124/mol.64.3.679

[49] Appl H, Holzammer T, Dove S, Haen E, Strasser A, et al (2011) Interactions of recombinant human histamine H_1_, H_2_, H_3_ and H_4_ receptors with 34 antidepressants and antipsychotics. Naunyn Schmiedebergs Arch Pharmacol 385: 145–170. 22033803 10.1007/s00210-011-0704-0

[50] Gibson-Berry KL, Whitin JC, Cohen HJ (1993) Modulation of the respiratory burst in human neutrophils by isoproterenol and dibutyryl cyclic AMP. J Neuroimmunol 43: 59–68. 8384637 10.1016/0165-5728(93)90075-a

[51] Barnett CC Jr, Moore EE, Partrick DA, Silliman CC (1997) Beta-adrenergic stimulation down-regulates neutrophil priming for superoxide generation, but not elastase release. J Surg Res 70: 166–170. 9245567 10.1006/jsre.1997.5118

[52] Hidaka H, Inagaki M, Kawamoto S, Sasaki Y (1984) Isoquinolinesulfonamides, novel and potent inhibitors of cyclic nucleotide dependent protein kinase and protein kinase C. Biochemistry. 23: 5036–5041. 10.1021/bi00316a032 6238627

[53] Rothermel JD, Jastorff B, Botelho LH (1984) Inhibition of glucagon-induced glycogenolysis in isolated rat hepatocytes by the Rp diastereomer of adenosine cyclic 3′,5′-phosphorothioate. J Biol Chem 259: 8151–8155. 6330102

[54] Lazarovici P, Rasouly D, Friedman L, Tabekman R, Ovadia H, et al (1996) K252a and staurosporine microbial alkaloid toxins as prototype of neurotropic drugs. Adv Exp Med Biol 391: 367–377. 8726076 10.1007/978-1-4613-0361-9_31

[55] Uemura T, Ohta Y, Nakao Y, Manaka T, Nakamura H, et al (2010) Epinephrine accelerates osteoblastic differentiation by enhancing bone morphogenetic protein signaling through a cAMP/protein kinase A signaling pathway. Bone 47: 756–765. 20637325 10.1016/j.bone.2010.07.008

[56] Kellenberger S, Muller K, Richener H, Bilbe G (1998) Formoterol and isoproterenol induce c-fos gene expression in osteoblast-like cells by activating β_2_-adrenergic receptors. Bone 22: 471–478. 9600780 10.1016/s8756-3282(98)00026-x

[57] Hoogland TM, Saggau P (2004) Facilitation of L-type Ca^2+^ channels in dendritic spines by activation of β_2_ adrenergic receptors. J Neurosci 24: 8416–8427. 15456814 10.1523/JNEUROSCI.1677-04.2004 PMC6729902

[58] Madden KS, Szpunar MJ, Brown EB (2011) β-Adrenergic receptors (β-AR) regulate VEGF and IL-6 production by divergent pathways in high β-AR-expressing breast cancer cell lines. Breast Cancer Res Treat 130: 747–758. 21234673 10.1007/s10549-011-1348-y PMC3126869

[59] Dostmann WRG, Taylor SS, Genieser HG, Jastorff B, Døskeland SO, et al (1990) Probing cyclic nucleotide binding sites of cAMP-dependent protein kinase I and II with analogs of adenosine 3′,5′-cyclic phosphorothioates. J Biol Chem 265: 10484–10491. 2162349

[60] Hourani SM, Boon K, Fooks HM, Prentice DJ (2001) Role of cyclic nucleotides in vasodilations of the rat thoracic aorta induced by adenosine analogues. Br J Pharmacol 2001 133: 833–40. 10.1038/sj.bjp.0704140 PMC1572848 11454656

[61] Seifert R, Lushington GH, Mou TC, Gille A, Sprang SR (2012) Inhibitors of membranous adenylyl cyclases. Trends Pharmacol Sci 33: 64–78. 22100304 10.1016/j.tips.2011.10.006 PMC3273670

[62] Kaukel E, Mundhenk K, Hilz H (1972) N^6^-monobutyryladenosine 3′:5′-monophosphate is the biologically active derivative of dibutyryladenosine 3′:5′-monophosphate in HeLa S3 cells. Eur J Biochem 27: 197–200. 4340276 10.1111/j.1432-1033.1972.tb01826.x

[63] Jackson EK, Raghvendra DK (2004) The extracellular cyclic AMP-adenosine pathway in renal physiology. Annu Rev Physiol 66: 571–599. 14977414 10.1146/annurev.physiol.66.032102.111604

[64] Russel FG, Koenderink JB, Masereeuw R (2008) Multidrug resistance protein 4 (MRP4/ABCC4): a versatile efflux transporter for drugs and signalling molecules. Trends Pharmacol Sci 29: 200–207. 18353444 10.1016/j.tips.2008.01.006

[65] Gloerich M, Bos JL (2010) Epac: defining a new mechanism for cAMP action. Annu Rev Pharmacol Toxicol 50: 355–375. 20055708 10.1146/annurev.pharmtox.010909.105714

[66] Seifert R, Dove S (2009) Functional selectivity of GPCR ligand stereoisomers: new pharmacological opportunities. Mol Pharmacol 75: 13–18. 19001067 10.1124/mol.108.052944

[67] Mukhopadhyay S, Howlett AC (2005) Chemically distinct ligands promote differential CB_1_ cannabinoid receptor-G_i_ protein interactions. Mol Pharmacol 67: 2016–2024. 15749995 10.1124/mol.104.003558

[68] Reher TM, Neumann D, Buschauer A, Seifert R (2012) Incomplete activation of human eosinophils via the histamine H_4_-receptor: Evidence for ligand-specific receptor conformations. Biochem Pharmacol 84: 192–203. 22516788 10.1016/j.bcp.2012.04.004

[69] Woo AY, Wang TB, Zeng X, Zhu W, Abernethy DR, et al (2009) Stereochemistry of an agonist determines coupling preference of β_2_-adrenoceptor to different G proteins in cardiomyocytes. Mol Pharmacol 75: 158–165. 18838481 10.1124/mol.108.051078 PMC2654765

[70] Carbonetti NH (2010) Pertussis toxin and adenylate cyclase toxin: key virulence factors of Bordetella pertussis and cell biology tools. Future Microbiol 5: 455–469. 20210554 10.2217/fmb.09.133 PMC2851156

[71] Orlic T, Loomis WH, Shreve A, Namiki S, Junger WG (2002) Hypertonicity increases cAMP in PMN and blocks oxidative burst by PKA-dependent and -independent mechanisms. Am J Physiol Cell Physiol 282: C1261–C1269. 11997240 10.1152/ajpcell.00479.2001

[72] Sedgwick JB, Berube ML, Zurier RB (1985) Stimulus-dependent inhibition of superoxide generation by prostaglandins. Clin Immunol Immunopathol 34: 205–215. 2981649 10.1016/0090-1229(85)90025-x

[73] Cronstein BN, Kramer SB, Rosenstein ED, Korchak HM, Weissmann G, et al (1988) Occupancy of adenosine receptors raises cyclic AMP alone and in synergy with occupancy of chemoattractant receptors and inhibits membrane depolarization. Biochem J 252: 709–715. 2844154 10.1042/bj2520709 PMC1149206

[74] Costantini TW, Deree J, Peterson CY, Putnam JG, Woon T, et al (2010) Pentoxifylline modulates p47phox activation and downregulates neutrophil oxidative burst through PKA-dependent and -independent mechanisms. Immunopharmacol Immunotoxicol 32: 82–91. 19839729 10.3109/08923970903183557

[75] Costa T, Klinz FJ, Vachon L, Herz A (1988) Opioid receptors are coupled tightly to G proteins but loosely to adenylate cyclase in NG108–15 cell membranes. Mol Pharmacol 34: 744–7454. 2849042

[76] Werner K, Schwede F, Genieser HG, Geiger J, Butt E (2011) Quantification of cAMP and cGMP analogs in intact cells: pitfalls in enzyme immunoassays for cyclic nucleotides. Naunyn Schmiedebergs Arch Pharmacol 384: 169–176. 21713381 10.1007/s00210-011-0662-6 PMC3145891

[77] Brodde OE, Michel MC (1995) Adrenergic and muscarinic receptors in the human heart. Pharmacol Rev 51: 651–690. 10581327

[78] Stallaert W, Dorn JF, van der Westhuizen E, Audet M, Bouvier M (2012) Impedance responses reveal β_2_-adrenergic receptor signaling pluridimensionality and allow classification of ligands with distinct signaling profiles. PLoS One 7: e29420. 22242170 10.1371/journal.pone.0029420 PMC3252315

[79] Liao CH, Lin SZ, Tseng CP, Day YJ, Chang CS, et al (2008) A benzodiazepines derived compound, 4-(3-chlorophenyl)-1,3-dihydronaphtho 2,3-b]1,4]diazepin-2-one (ND700C), inhibits fMLP-induced superoxide anion release by activating protein phosphatase 2A in human neutrophils. Biochem Pharmacol 76: 1728–1739. 18823951 10.1016/j.bcp.2008.09.002

[80] Liu FC, Day YJ, Liou JT, Yu HP, Liao HR (2012) Splitomicin inhibits fMLP-induced superoxide anion production in human neutrophils by activate cAMP/PKA signaling inhibition of ERK pathway. Eur J Pharmacol 688: 68–75. 22634165 10.1016/j.ejphar.2012.05.006

[81] Kaya AI, Onaran HO, Ozcan G, Ambrosio C, Costa T, et al (2012) Cell contact-dependent functional selectivity of β_2_-adrenergic receptor ligands in stimulating cAMP accumulation and extracellular signal-regulated kinase phosphorylation. J Biol Chem 287: 6362–6374. 22241475 10.1074/jbc.M111.301820 PMC3307305

[82] Beste KY, Seifert R (2013) cCMP, cUMP, cTMP, cIMP and cXMP as possible second messengers: development of a hypothesis based on studies with soluble guanylyl cyclase α_1_β_1_ . Biol Chem 394: 261–270. 23087103 10.1515/hsz-2012-0282

[83] Uzkeser H, Cadirci E, Halici Z, Odabasoglu F, Polat B, et al (2012) Anti-inflammatory and antinociceptive effects of salbutamol on acute and chronic models of inflammation in rats: involvement of an antioxidant mechanism. Mediators Inflamm 2012: 438912. 22665951 10.1155/2012/438912 PMC3361306

[84] Sun Y, Huang J, Xiang Y, Bastepe M, Juppner H, et al (2007) Dosage-dependent switch from G protein-coupled to G protein-independent signaling by a GPCR. EMBO J 26: 53–64. 17170700 10.1038/sj.emboj.7601502 PMC1782364

