# Supplementary figures and images for: Dissociations in the Effects of β2-Adrenergic Receptor Agonists on cAMP Formation and Superoxide Production in Human Neutrophils: Support for the Concept of Functional Selectivity
Source: PLoS One. 2013 May 31;8(5):e64556. doi: 10.1371/journal.pone.0064556 (PMC3669315; doi:10.1371/journal.pone.0064556)

## Slide 1
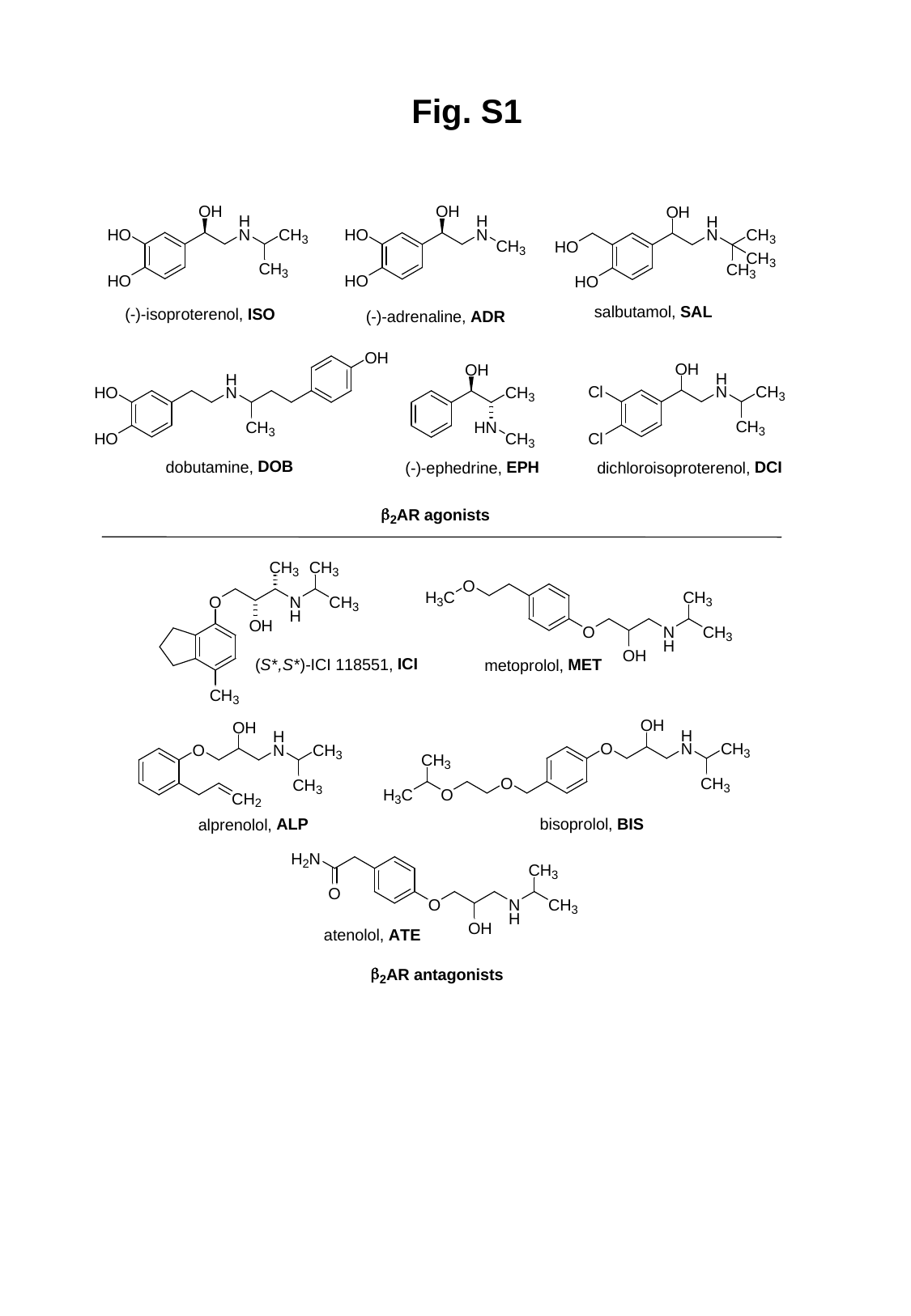

Fig. S1

Supplement: Figure S1 — Structures of the β2AR agonists and antagonists examined in this study. (PPTX) [file pone.0064556.s001.pptx]

## Slide 1
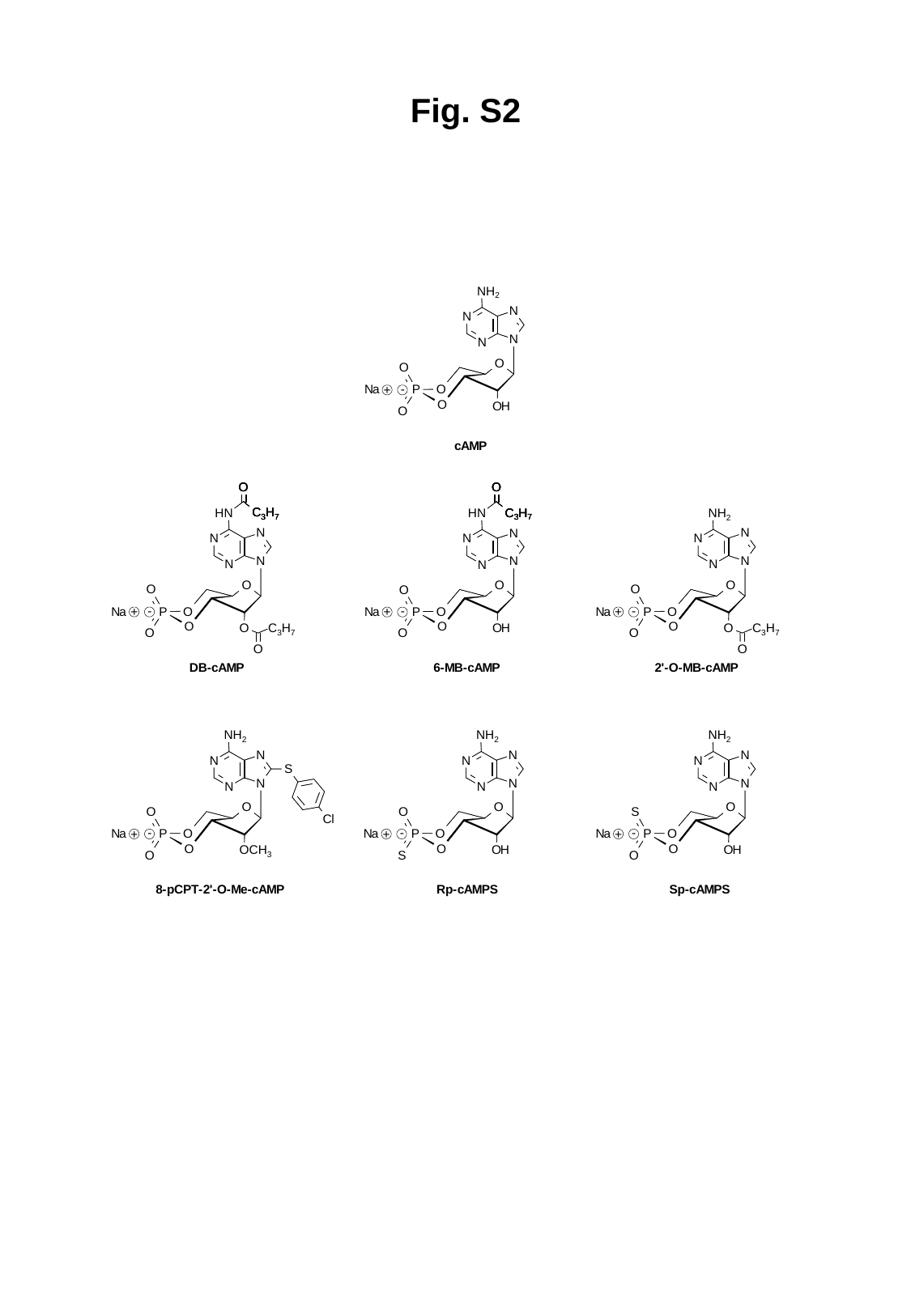

Fig. S2

Supplement: Figure S2 — Structures of cAMP and cAMP analogs examined in this study. (PPTX) [file pone.0064556.s002.pptx]
